# Predicted novel hypertrehalosaemic peptides of cockroaches are verified by mass spectrometry

**DOI:** 10.1007/s00726-023-03337-7

**Published:** 2023-10-26

**Authors:** Heather G. Marco, Simone König, Gerd Gäde

**Affiliations:** 1 Department of Biological Sciences, University of Cape Town, Rondebosch, Cape Town, South Africa; 2 IZKF Core Unit Proteomics, University of Münster, Münster, Germany

**Keywords:** Cockroaches, Blaberidae, Hypertrehalosaemic peptides, Primary sequence elucidation, Mass spectrometry, Hydroxyproline modification, Molecular evolution

## Abstract

Small neuropeptides from the corpora cardiaca are responsible in cockroaches for the mobilisation of trehalose from the fat body into the haemolymph. Such hypertrehalosaemic hormones (HrTHs) belong to the large family of insect adipokinetic hormones (AKHs); a few HrTHs were previously sequenced from cockroaches, and from genomic and/or transcriptomic information one may predict the genes encoding HrTHs from more species. Definite elucidation of the primary structure of the mature peptide with putative modifications needs analytical chemical methods. In the current study, we use high-resolution mass spectrometry coupled with liquid chromatography to identify unequivocally the HrTHs of 13 cockroach species. Either genomic/transcriptomic information was available for most of the species examined, or from related species. We confirm predicted novel sequences and find hydroxyproline modification for the majority of the peptides. The novel decapeptides are structurally close to Bladi-HrTH, which is found in all seven of the investigated blaberid subfamilies. Bladi-HrTH and all the novel peptides elicit a hypertrehalosaemic response in *Periplaneta americana*, a blattid cockroach.

**Supplementary Information:**

The online version contains supplementary material available at 10.1007/s00726-023-03337-7.

## Introduction

The presence of a factor in the corpora cardiaca (CC) of the American cockroach *Periplaneta americana* that was able to elevate haemolymph sugar levels was first published by Steele (1961). The factor was called “hyperglycaemic” but later rather termed “hypertrehalosaemic factor or hormone” (see Goldsworthy and Gäde 1983), because it is the disaccharide trehalose that is the main circulating sugar in most insects. Indeed, an increase of trehalose was shown in *P. americana* upon hormonal injection by means of isocratic high-performance liquid chromatography (HPLC) on an amino phase support and refractor index detection (Gäde 1991a). Various attempts were made by different research groups to isolate hypertrehalosaemic hormone (HrTH) of this blattid cockroach (see Goldsworthy and Gäde 1983), and in 1984, two absorbance peaks with hypertrehalosaemic activity were separated from extracts of *P. americana* CC, and the amino acid composition of the respective octapeptides was determined (Gäde 1984, 1985a). Parallel to these studies, peptides with identical amino acid composition had been isolated from *P. americana* CC using a skeletal muscle bioassay to guide the separation (O’Shea et al. 1984). The myoactive peptides, then called MI and MII, were sequenced (Witten et al. 1984), and the synthetic compounds compared well in HPLC retention time and biological activity with the two hypertrehalosaemic peptides (Gäde 1985b). A third research group came to the same sequence results using a metabolic (= hyperglycaemic) and myotropic (= cardioacceleratory) bioassay during the separation procedure (Scarborough et al. 1984). In 1986, the primary structures for the hypertrehalosaemic peptides of *P. americana* were confirmed (Siegert and Mordue 1986); today, they are code-named with the acronyms Peram-CAH-I and Peram-CAH-II, with reference to the cardioacceleratory hormone function. In the same year, a decapeptide hormone, now known as Bladi-HrTH, was isolated and sequenced from two blaberid cockroaches *Blaberus discoidalis* (Hayes et al. 1986) and *Nauphoeta cinerea* (Gäde and Rinehart 1986; Gäde 1987). In the cockroach *Polyphaga aegyptiaca,* two octapeptide hypertrehalosaemic peptides were sequenced from the CC: one was previously known to occur in the glands of the tenebrionid beetle *Tenebrio molitor* (Gäde and Rosinski 1990) and was therefore called Tenmo-HrTH; the second peptide, however, had a unique sequence and is known as Polae-HrTH (Gäde and Kellner 1992). Later, these five peptides were confirmed in species from most taxonomic families of the cockroaches (Gäde 1989; Gäde and Rinehart 1990; Veenstra and Camps 1990; Gäde et al. 1997; König et al. 2005; Predel and Gäde 2005; Roth et al. 2009; Zeng et al. 2021; Jiang et al. 2023).

These HrTHs are members of the well-known adipokinetic hormone (AKH) peptide family. Physiological studies on the effect of HrTHs in cockroaches were mainly performed on *P. americana*, as well as *N. cinerea, B. discoidalis,* and *Blaptica dubia,* and included experiments on the release of the peptides, activation of adenylate cyclase and glycogen phosphorylase, structure–activity relations, and investigations into the influence on the regulation of glycolysis (see, e.g., Gäde 1985c, 1986, 1988, 1991b, 1992; Ford and Hayes 1988; Hayes and Keeley 1990; Sevala and Steele 1991; Gäde and Hayes 1995; Keeley et al. 1996; Becker and Wegener 1998).

As early as 1989 (Gäde 1989), the primary structures of cockroach hypertrehalosaemic peptides have been used to speculate on and/or confirm phylogenetic relatedness of cockroach (order: Blattodea) species. For the first time in 2009, mainly CAPA peptides (insect neuropeptides that are encoded by the *capability* gene [Kean et al. 2002]), but also hypertrehalosaemic peptides and sulfakinins were used in a proteomic approach to reconstruct the phylogenetic relationships of cockroaches (Roth et al. 2009). A cladogram constructed from the sequence data of these neuropeptides agreed quite well with analyses which took molecular and morphological data into account (for example, Inward et al. 2007; Klass and Meier 2006). The most recent phylogenomic analysis of Blattodea was published by Evangelista et al. 2019. The topologies of that study were mainly confirmed by Bläser et al. (2020) who used the precursor sequences of 17 neuropeptides but not the ones of the AKH peptide family. Recently, Jiang et al. (2023) confirmed, in general, the phylogeny of Blattodea as outlined by Evangelista et al. (2019) with the sequence data of the HrTH receptor, whereas the use of HrTH precursor data did not achieve such a good phylogenetic fit. This most recent study also revealed an early HrTH gene duplication event in the ancestor of one of the superfamilies, the Blaberoidea; based on transcriptomic/genomic data, seven novel sequences are suggested for decapeptides of the AKH peptide family in various blaberoid species.

Bioinformatic predictions, however, are not sufficient to elucidate the structure of a mature AKH. Post-translational modifications are not encoded, and the presence of a gene is not a given fact of expression in the organism. Thus, to arrive at definitive information on the primary sequence of an AKH in any given species, it is imperative to isolate the peptide from the insect and validate its sequence using analytical chemistry methods. In the current study, we used high-resolution mass spectrometry (MS) coupled with nanoflow reversed-phase liquid chromatography (RP-LC) to investigate HrTH peptides from 13 cockroach species. For six species, peptide sequences were available in genomic databases; these were candidates for novel decapeptides. We also included some species with known HrTHs as controls. To examine biological activity of the novel decapeptides, an in vivo metabolic biological assay was performed with adult *P. americana* specimens as acceptor insect.

## Materials and methods

### Insects

Adult specimens of various cockroach species were used in this study: the species and their taxonomic affiliations are listed in Table 1; no regard was paid to sex and age of the specimens. All species were purchased from commercial breeders with exceptions. Adult males of the American cockroach, *P. americana*, were a gift from the research group of Prof. R. Predel (University of Cologne, Germany) and *Anaplecta* ssp and *Xestoblatta cavicola* from the research group of Prof. P. Deleporte (University of Rennes, France). *Aptera fusca* was caught on the premises of the University of Cape Town, South Africa.

**Table 1:**
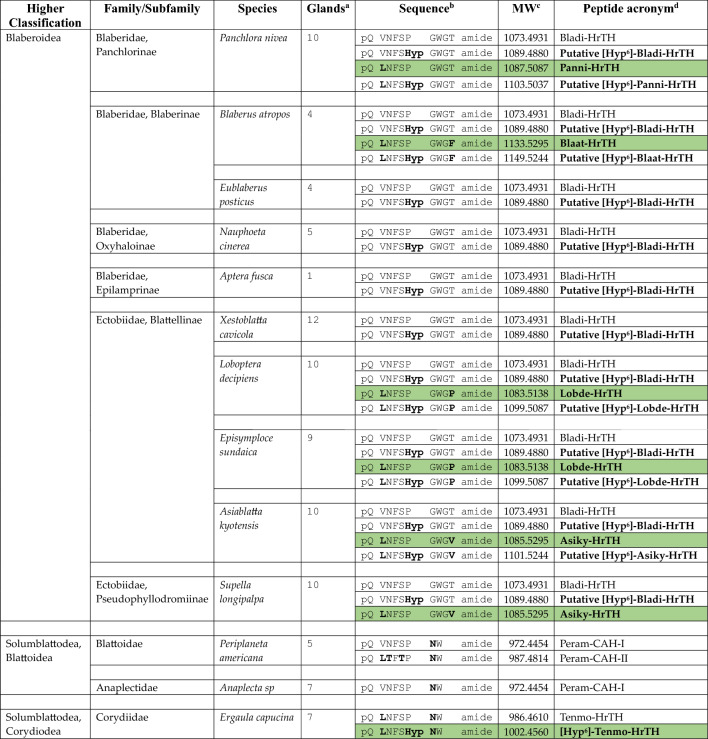
Hypertrehalosaemic hormones (HrTHs) of Blattodea species determined in this study using high-resolution MS

The taxonomic classification is as per Evangelista et al. (2019)

^a^The number of dissected corpora cardiaca used in the MS analyses

^b^Gaps are introduced to better align similarities between octapeptides and decapeptides. Amino acid substitutions with respect to Bladi-HrTH are shown in bold letters. Substantiated novel decapeptide sequences (predicted from molecular databases, Jiang et al. 2023) are highlighted in green

^c^*MW* molecular weight

^d^Novel peptides (including those with the hydroxyproline modification) are indicated by their acronyms in bold text (last column). Note, putative refers to peptide sequences still to be validated by a corresponding synthetic peptide

The American cockroach was used for biological assays (see below); the animals were held at 25 ± 2 °C, RH at 60%, and 14-h light: 10-h dark cycle and fed with a mixture of dog and rabbit food plus water ad libitum.

### Tissue preparation and peptide isolation

CCs were dissected from individual cockroaches of the various species immediately after arrival from the supplier or commercial dealer with the aid of a stereomicroscope at 20- to 40-fold magnification. The glands from the same species were pooled in a microcentrifuge tube containing 80% methanol, extracted by approved methods (Gäde 1984), and dried in a vacuum centrifuge, and aliquots were used in biological assays with the American cockroach and for MS.

### Biological assay

The dried methanolic CC extracts were reconstituted in distilled water. Synthetic peptides were dissolved in 40 µL of 50/50 *v/v* methanol and 0.1% formic acid containing 5% acetonitrile and then diluted 1:1000 with distilled water for injection into *P. americana* to measure their hypertrehalosaemic activity as previously outlined (Gäde 1980). Briefly, experimentation took place at 25 ± 2 °C; cockroaches were put into individual film canisters with a wad of moist cotton wool and left to rest in the dark for 1 h before 1 µL of haemolymph was withdrawn from the base of a leg with a glass microcapillary. The haemolymph was blown into a glass test tube containing 100 µL sulfuric acid. The animal was then injected into the abdominal cavity with 10 µL of the test solution via a 25 µL Hamilton syringe and returned to rest in the canister for 90 min whereafter a second sample of haemolymph was removed as described above.

The colorimetric measurement of carbohydrates was done according to Holwerda et al. (1977): 1 mL of anthrone solution (120 mg anthrone, 60 mL of H_2_SO_4_, 30 mL of distilled water) was added to each test tube, mixed thoroughly, and then heated at 100 °C for 8 min in a heating block. Thereafter, tubes were cooled in cold water for 5 min and incubated in the dark for 30 min, and the samples were measured in a Helios spectrophotometer at a wavelength of 585 nm. The carbohydrate concentration could be gleaned from a calibration curve for carbohydrates using the same methodology with known amounts of glucose.

The difference in carbohydrate concentration before and after injection was calculated for each individual animal, and a paired t-test was used for calculating statistical significance in Excel. An analysis of variance (ANOVA) with Tukey’s HSD (honestly significant difference) test (to control the type I error rate, see Fernandez 1992) was used to test for significant differences between different groups of cockroaches injected with different synthetic peptides at the same dose (10 pmol). Briefly, the distribution of data, including an assessment of normality, was carried out with the UNIVARIATE Procedure to validate the use of ANOVA; the relative per cent change following injection of a substance was calculated by the formula ((T90 – T0) / T0) *100, where the carbohydrate concentration before injection (T0) and 90 min after injection (T90) is considered for each substance. The mean relative per cent change for each group of animals was compared by Tukey’s HSD test (p ≤ 0.05). The normality test, ANOVA, and Tukey’s test were generated using SAS Enterprise Guide v. 8.3 (SAS Institute, Cary, NC, USA).

### AKH separation and sequence characterisation by LC–MS

The dried methanolic CC extracts were dissolved in 10 μL methanol followed by 10 μL 0.1% formic acid containing 5% acetonitrile. For LC–MS/MS, Synapt G2 Si (Q-TOF with ion mobility function) coupled to M-Class nano-UPLC (Waters Corp., Manchester, UK) was employed using C18 μPAC columns (trapping and 50 cm analytical; PharmaFluidics, Ghent, Belgium) with a 30 min gradient (10–60%; solvent system 100% water versus 100% acetonitrile, both containing 0.1% formic acid; 1 μL injection volume). AKH candidates were identified by target-MS (MS/MS on pre-selected *m/z* values) for eligible known peptide masses from related insect species using their singly and doubly charged ions, as well as by screening with low/high collision energy switching for the gas phase loss of the tryptophan immonium ion in data-independent runs. Moreover, AKH candidates were obtained by manual interrogation of data-dependent runs and the use of marker fragment ions discovered for proline-containing AKHs (König et al. 2023).

Sequence ion assignment was used as calculated by the MassLynx spectrometer software, which treats pyroglutamate (Pyr) as terminal modification rather than a modified amino acid, thus creating a label shift for ion assignment by one in comparison to the amino acid number. The fragment ion tables for the spectra shown here are available in the *Supplement* for clarification. Peptide sequences were validated by comparison to the performance of the respective synthetic peptides; in the case of the hydroxyproline forms where synthetic peptides were mostly not available, the sequences could not be validated and those peptides are considered putative. Both the endogenous and the synthetic samples were spiked with bradykinin 1–7 (Sigma, 1 pmol/µL stock solution) for control of the retention time, which was about 6 min earlier than for the AKHs. It allowed correction of the LC arrival time following heavy unrelated use of the instrumentation, if necessary. The peptides were run with identical parameters separated by blank runs.

### Synthetic peptides

The novel adipokinetic peptides elucidated in this study, scrambled Peram-CAH-I, Tenmo-HrTH, and its [Hyp^6^] form, were purchased from Pepmic Co., Ltd. (Suzhou, China). Peram-CAH-I and Bladi-HrTH were purchased from Peninsula Laboratories (Belmont, CA, USA).

## Results

### Hypertrehalosaemic activity of cockroach CC extracts

In a first series of experiments, glandular extracts from the various cockroaches under study were tested in mobilising trehalose in American cockroaches. CC material from a subset of cockroaches only was analysed in this study: some we had examined before (*S*. *longipalpa, N. cinerea*, Gäde 1989) and are not repeated here, other species were not available in sufficient numbers (*Aptera fusca*), or not easily obtained. We further selected to study mainly those species of whom we had genomic/transcriptomic data that hinted at novel HrTHs not previously identified in CC extracts.

As summarised in Table 2, 0.2 gland equivalents of the CC were able to elevate the level of total carbohydrates in the haemolymph significantly, whereas control injections with water had no significant effect. The total increase in circulating carbohydrates in *P. americana* 90 min after injection of the conspecific CC extracts was in the same range (15 to 18 µg/µL) as measured after injection of the positive control, i.e. the synthetic peptide Peram-CAH-I, an endogenous octapeptide of *P. americana* (Table 2). The effect of the CC extract of *A. kyotensis*, however, was slightly lower (Table 2), and this may be due to the smaller size of the insect and its CC. Nevertheless, this experiment has clearly demonstrated the presence of hypertrehalosaemic material in the CC of the selected cockroaches.

**Table 2:** Biological activity of crude methanolic extracts of corpora cardiaca (CC) from various cockroaches in an in vivo bioassay with the American cockroach, *Periplaneta americana*

Treatment	*n*	[Carbohydrates]T_0_min (µg/µL)	[Carbohydrates]T_90_min (µg/µL)	Difference (µg/µL)	P*
Distilled water	8	16.80 ± 3.93	16.20 ± 4.01	− 0.60 ± 2.75	NS
*P. nivea* (0.2 gland pair equivalent)	5	18.17 ± 2.00	33.03 ± 6.82	14.86 ± 8.16	0.008
*B. atropos* (0.2 gland pair equivalent)	6	18.80 ± 5.86	37.14 ± 8.91	18.34 ± 6.73	0.0006
*E. posticus* (0.2 gland pair equivalent)	5	21.92 ± 2.00	38.30 ± 8.39	16.37 ± 7.20	0.004
*E. sundaica* (0.2 gland pair equivalent)	5	20.60 ± 2.30	39.23 ± 2.15	18.63 ± 3.91	0.0002
*A. kyotensis* (0.2 gland pair equivalent)	5	18.86 ± 2.61	31.04 ± 5.95	12.18 ± 3.40	0.0007
Peram-CAH-I (10 pmol)	7	18.14 ± 2.06	34.59 ± 4.107	16.45 ± 3.43	0.00001

CC extracts were derived from *Panchlora nivea*, *Blaberus atropos*, *Eublaberus posticus*, *Episymploce sundaica,* and *Asiablatta kyotensis*. Distilled water and the synthetic equivalent of an endogenous *P. americana* HrTH peptide were injected as control substances

Data given as mean ± SD

^*^A paired *t*-test was applied to compare data before and after injection in the same individuals. *NS* not significant

### LC–MS sequence analysis of hypertrehalosaemic peptides from various cockroaches

**Fig. 1:**
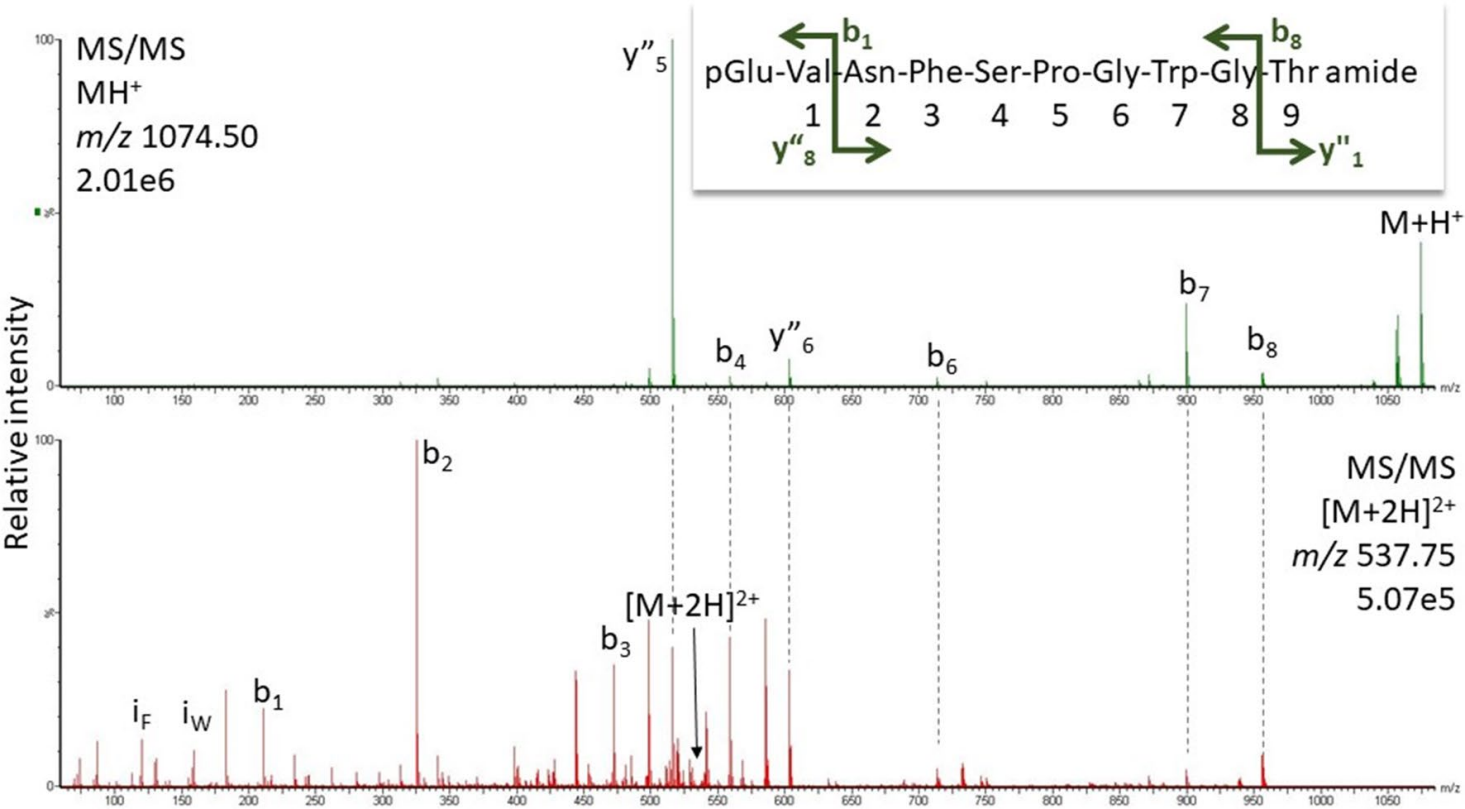
HYPERLINK "sps:id::fig1||locator::gr1||MediaObject::0"MS target analysis for Bladi-HrTH in *Panchlora nivea* (for validation, see Supplementary Figure S2A). The singly and the doubly charged ions were fragmented (M + H^+^ *m/z* 1074.50, [M + 2H].^2+^ *m/z* 537.75). Peaks were labelled according to the b- and y-ion series as calculated in Supplementary Figure S1

In most of the investigated species, the decapeptide Bladi-HrTH (pQVNFSPGWGT amide) was detected (Table 1; for an exemplary spectrum, from the CC of *P. nivea*, see Fig. 1) and validated (see Supplementary Information [SI], Figs. S1, S2). Interestingly, for Bladi-HrTH itself, as well as its novel decapeptide relatives (see below), hydroxyproline-containing forms (Hyp) were found in LC and sequenced by MS/MS (Table 1), but in most cases, this was not validated with a synthetic peptide and hence termed here as “putative”. Exemplary spectra of Hyp-modified-HrTHs are shown below for *P. nivea* and *Ergaula capucina.*

### The Blaberoidea

#### *Eublaberus posticus*, *Nauphoeta cinerea*, *Aptera fusca*, *Xestoblatta cavicola*

For four species of the Blaberidae, Bladi-HrTH, and its Hyp derivative were detected and sequenced (Table 1).

#### *Panchlora nivea*

**Fig. 2:**
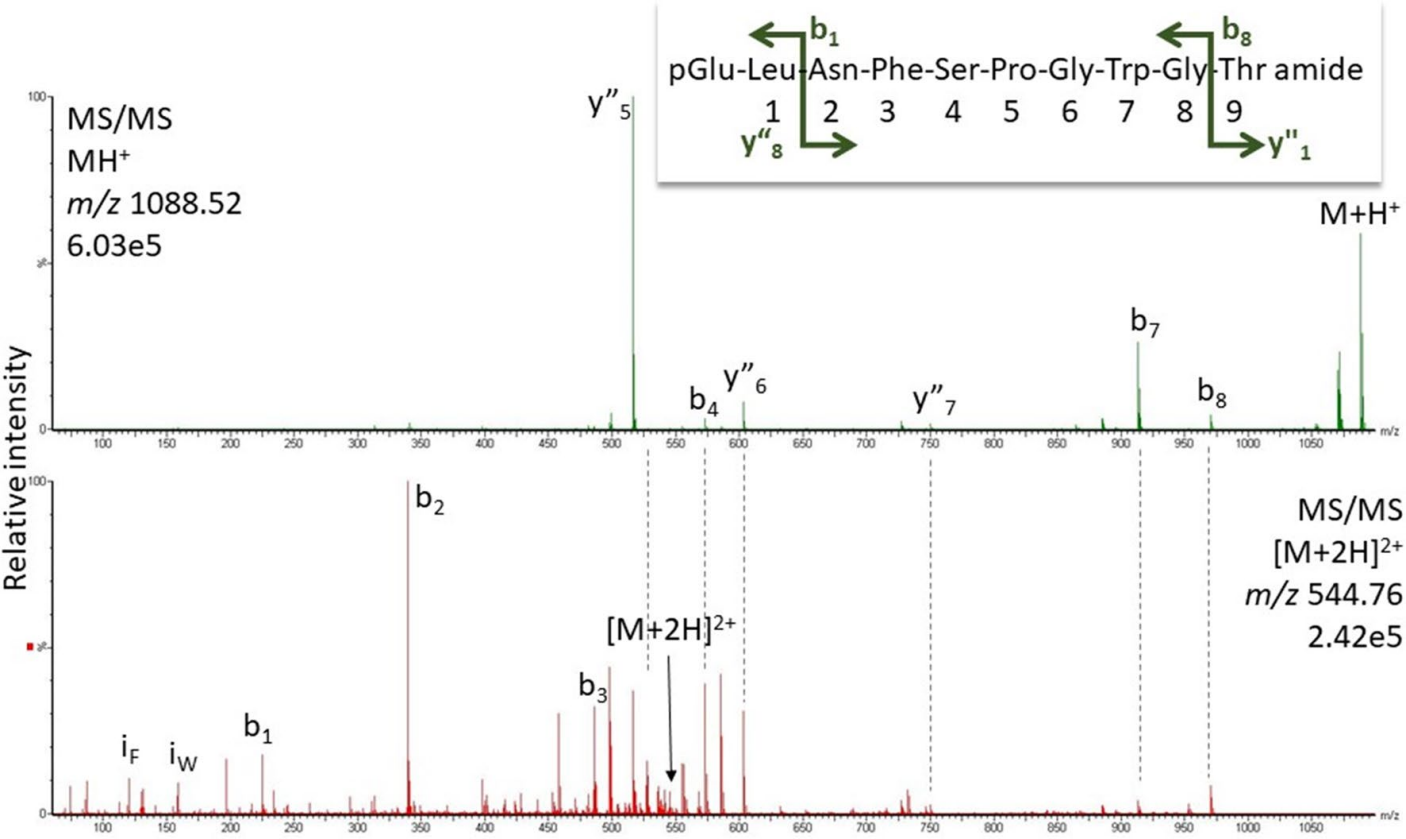
MS target analysis for L^2^ Bladi-HrTH (= Panni-HrTH) in *Panchlora nivea* (for validation, see Supplementary Figure S4). The singly and the doubly charged ions were fragmented (M + H^+^ *m/z* 1088.52, [M + 2H]^2+^, *m/z* 544.76). Peaks were labelled according to the b- and y-ion series as calculated in Supplementary Figure S3. This is a novel member of the AKH family

**Fig. 3:**
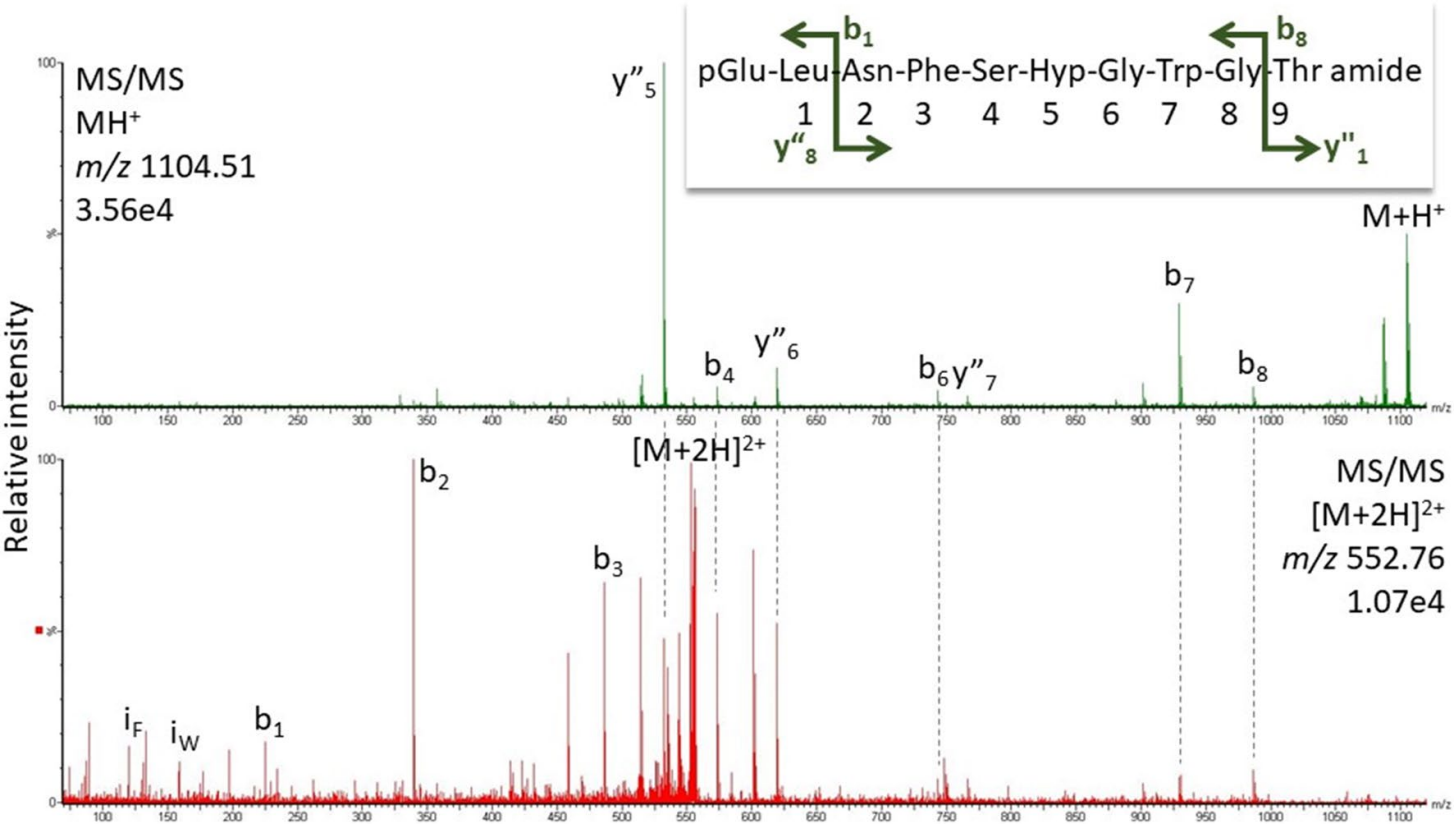
MS/MS analysis for the singly and the doubly charged peptide ions (*m/z* 1104.51 and 552.76) of a peptide assigned to [Hyp^6^]-Panni-HrTH in *Panchlora nivea* CC extract. Peaks were labelled according to the b- and y-ion series as calculated in Supplementary Figure S5. For original spectra, see Figure S6. The shift of 16 Da for the abundant fragment ions around the Pro residue (y”_5_, y”_6_, y”_7_, b_7_, b_8_) proves the position of the oxidation on the Pro residue

Besides Bladi-HrTH (Fig. 1) and its Hyp derivative, another AKH was detected with a sequence of pQLNFSPGWGT amide (Fig. 2). This novel, predicted peptide, now code-named Panni-HrTH, eluted ~ 2 min later than Bladi-HrTH (33.8 min) in LC and is, structurally, a Leu^2^ modified version of Bladi-HrTH. The sequence of Panni-HrTH was validated using the synthetic peptide (SI, Fig. S3, S4). A Hyp variant of Panni-HrTH had a retention time of 32.2 min, and a putative sequence was assigned on the basis of MS/MS sequencing (Fig. 3; SI Fig. S5, S6).

#### *Blaberus atropos*

**Fig. 4:**
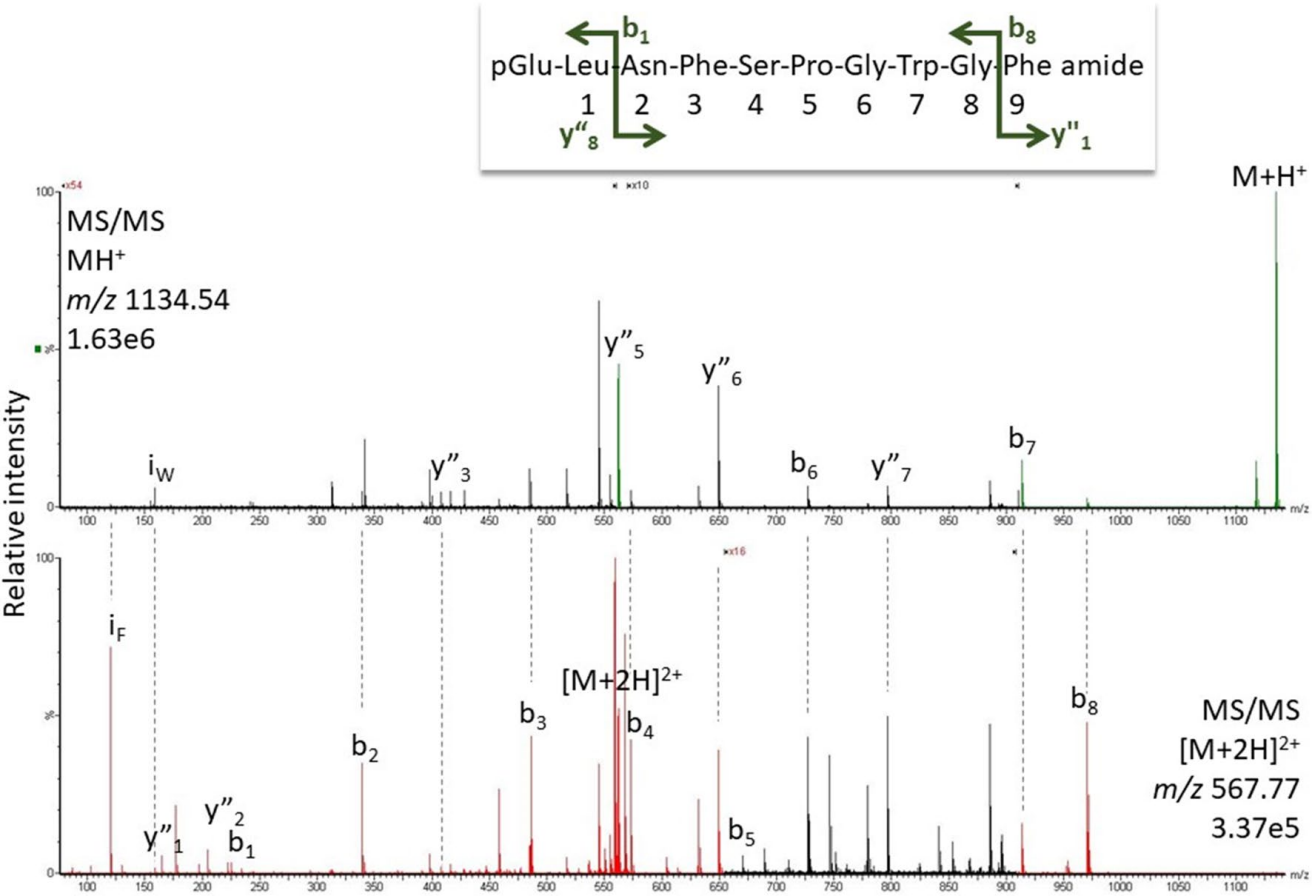
MS target analysis for L^2^F^10^ Bladi-HrTH (= Blaat-HrTH) in *Blaberus atropos* (for validation, see Supplementary Figures S7 and S8). The singly and the doubly charged ions were fragmented (M + H^+^ *m/z* 1134.54, [M + 2H]^2+^ *m/z* 567.77). Peaks were labelled according to the b- and y-ion series as calculated in Supplementary Figure S7. This is a novel member of the AKH family

Bladi-HrTH and a Hyp derivative of Bladi-HrTH were detected in the CC extract of *B. atropos* (Table 1) along with a novel decapeptide with the primary sequence of pQLNFSPGWGF amide (37.7 min, Fig. 4). This novel sequence was genomically predicted, and the peptide is here given the code-named Blaat-HrTH and validated. Compared with Bladi-HrTH, Blaat-HrTH has a Leu^2^ and Phe^10^ modification (Table 1); the sequence was validated with a corresponding synthetic peptide (SI, Fig. S7, S8). A Hyp variant of Blaat-HrTH was also sequenced.

#### *Loboptera decipiens* and *Episymploce sundaica*

**Fig. 5:**
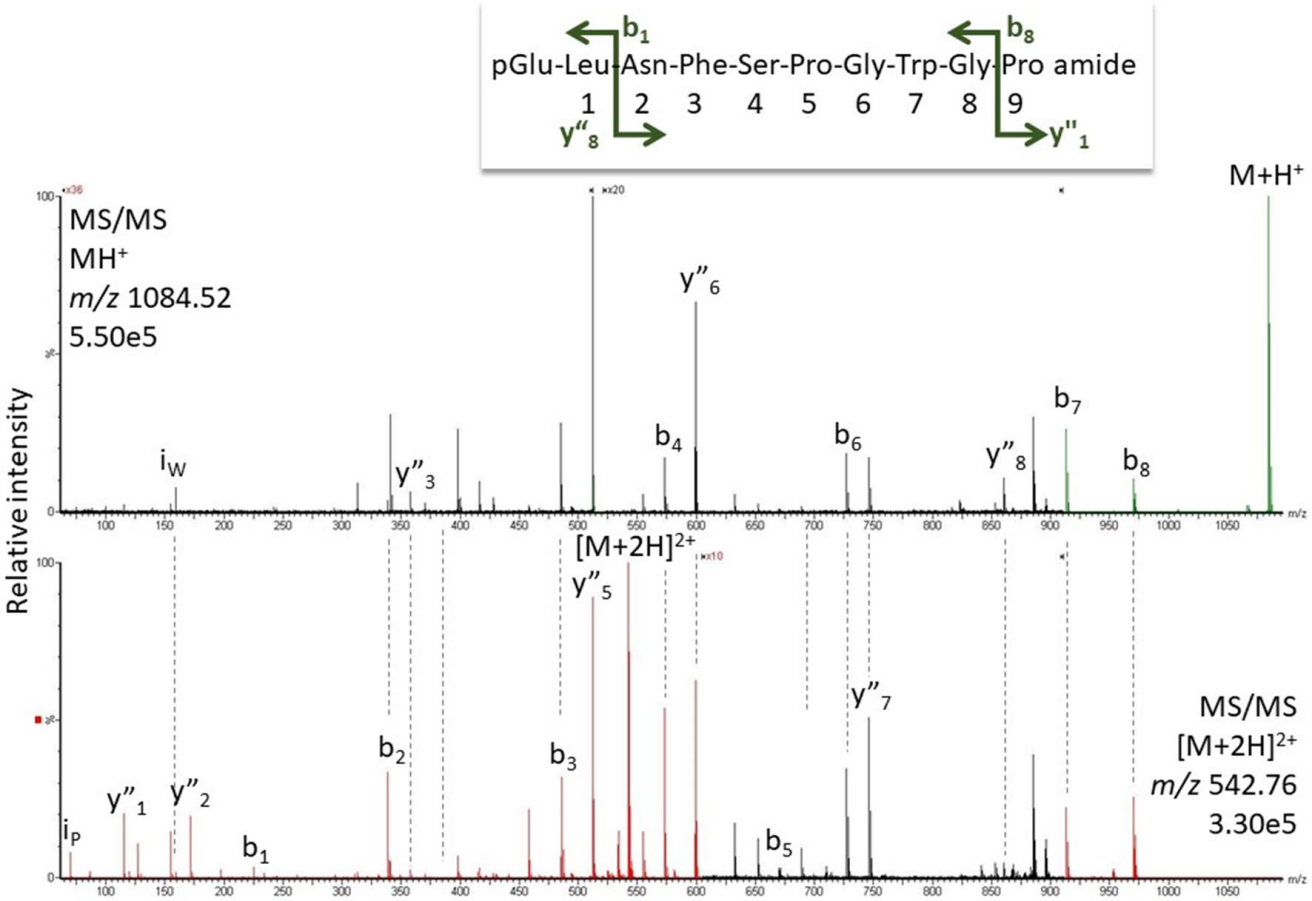
MS target analysis for L^2^P^10^ Bladi-HrTH (= Lobde-HrTH) in *Loboptera decipiens* (for validation, see Supplementary Figure S10). The singly and the doubly charged ions were fragmented (M + H^+^ *m/z* 1084.52, [M + 2H]^2+^ *m/z* 542.76). Peaks were labelled according to the b- and y-ion series as calculated in Supplementary Figure S9. This is a novel member of the AKH family

Identical peptides were found in *L. decipiens* and *E. sundaica*: Bladi-HrTH and its Hyp derivative, as well as a novel decapeptide sequence (pQLNFSPGWGP amide, 34.1 min, Fig. 5) and a Hyp variant of the latter (Table 1). The novel sequence was predicted from genomic information and first detected here in *L. decipiens*; hence, we assign the code-named Lobde-HrTH for the novel peptide that is common to both *L. decipiens* and *E. sundaica*. Compared with Bladi-HrTH, Lobde-HrTH has a Leu^2^ and a Pro^10^ substitution; the primary sequence was validated (SI, Fig. S9, S10).

#### *Asiablatta kyotensis* and *Supella longipalpa*

**Fig. 6:**
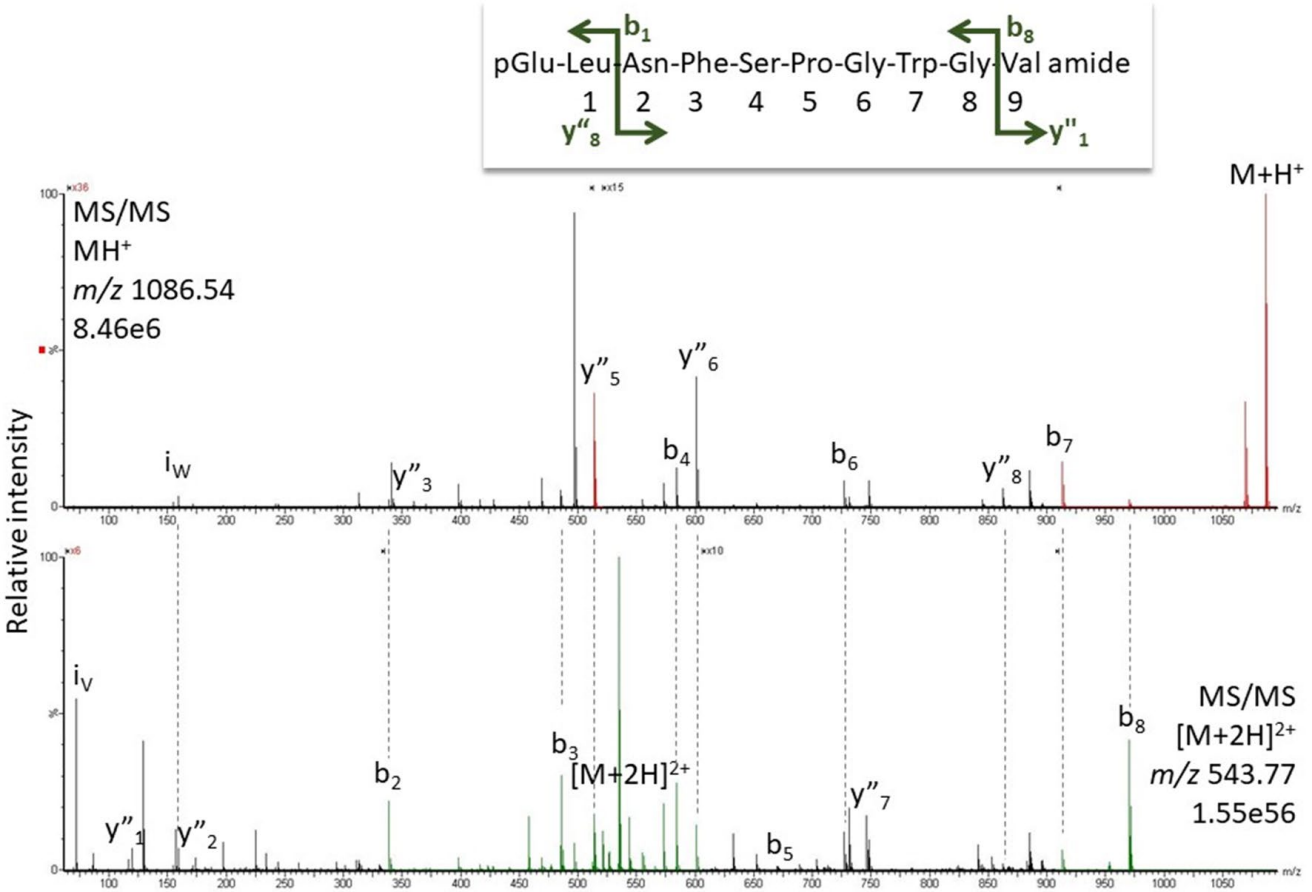
MS target analysis for L^2^V^10^ Bladi-HrTH (= Asiky-HrTH) in *Asiablatta kyotensis* (for validation, see Supplementary Figure S12). The singly and the doubly charged ions were fragmented (M + H^+^ *m/z* 1086.54, [M + 2H]^2+^ *m/z* 543.77). Peaks were labelled according to the b- and y-ion series as calculated in Supplementary Figure S11. This is a novel member of the AKH family

In both species, Bladi-HrTH and its Hyp derivative were found in the CC extracts, plus a novel decapeptide (pQLNFSPGWGV amide, 35.6 min, Fig. 6; Table 1). This novel peptide was first biochemically demonstrated here in *A. kyotensis* and is, therefore, given the code-named Asiky-HrTH. The structure was predicted from genomic information and was validated with a corresponding synthetic peptide (SI, Fig. S11, S12). In comparison to Bladi-HrTH, Asiky-HrTH has two modifications (Leu^2^ and Val^10^). Curiously, the Hyp variant of Asiky-HrTH was only detected and sequenced from *A. kyotensis* and not from *S. longipalpa* CC (Table 1)—this may possibly be due to a low concentration (below detection) in the very tiny CC of *S. longipalpa*.

### The Solumblattodea

#### *Periplaneta americana*, *Anaplecta ssp,* and *Ergaula capucina*

**Fig. 7:**
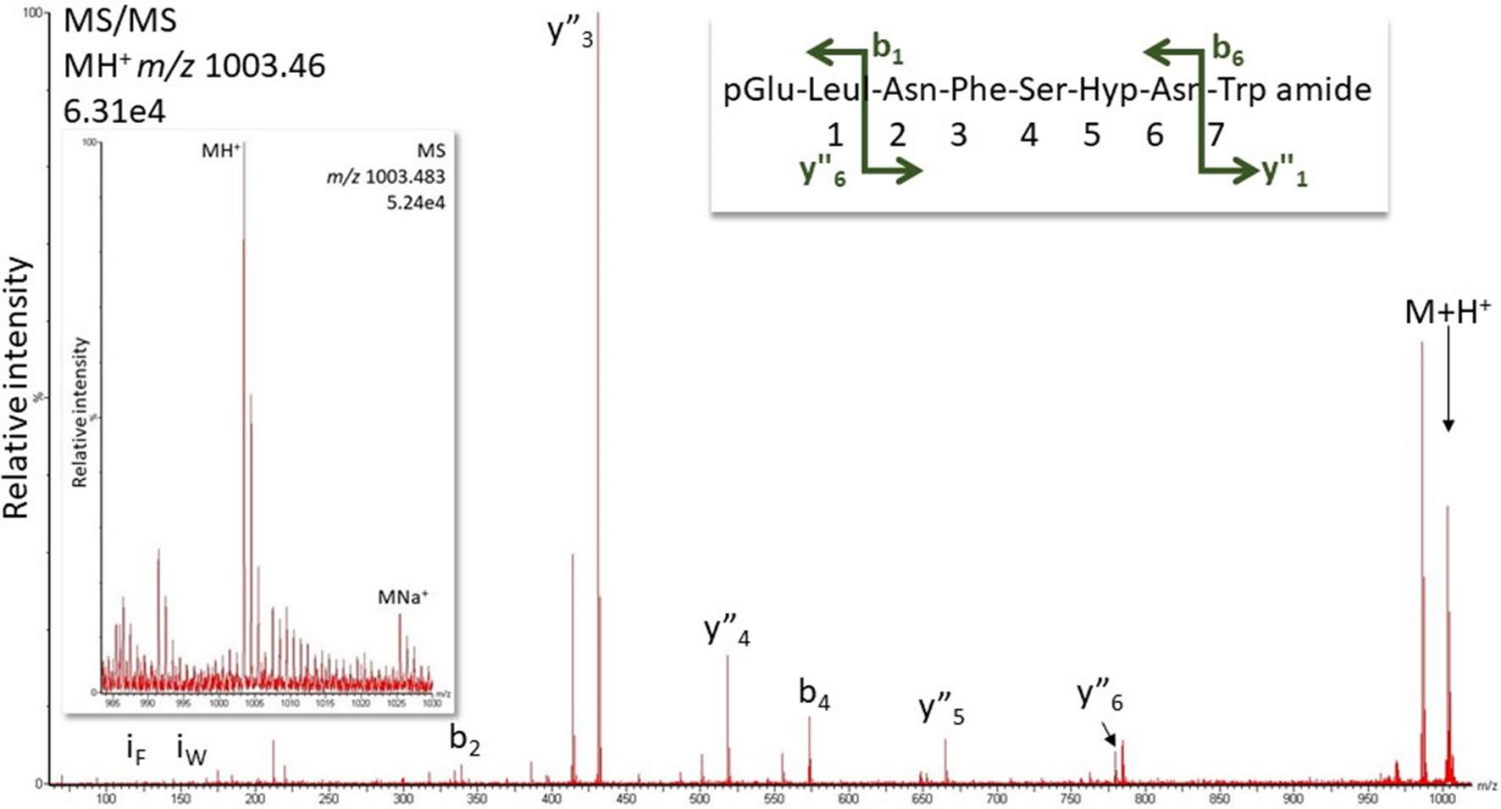
MS/MS analysis for [Hyp^6^]-Tenmo-HrTH in *Ergaula capucina* using the singly-charged peptide ion (*m/z* 1003.46). Peaks were labelled according to the b- and y-ion series as calculated in Supplementary Figure S17. The shift of 16 Da for the abundant fragment ions around the Pro residue (y”_3_, y”_4_, y”_5_, y”_6,_ b_4_) proves the position of the oxidation on the Pro residue. For original spectrum and validation with the synthetic compound, see Figure S18. Inset: overview spectrum showing the protonated and sodiated peptide peaks

*P. americana* is known to contain the two hypertrehalosaemic octapeptides Peram-CAH-I (pQVNPSPNW amide) and Peram-CAH-II (pQLTFTPNW amide) (see Introduction for references; SI, Fig. S13). We also detected these two octapeptides (32.1 min, 34.9 min) and show the spectra in Supplementary Fig. S14. In *Anaplecta ssp,* only Peram-CAH-I was found (SI, Fig. S14), while *E. capucina* presented the well-known octapeptide Tenmo-HrTH (pQLNFSPNW amide) (SI, Fig. S15, S16) and the Hyp form of Tenmo-HrTH (Fig. 7). [Hyp^6^]-Tenmo-HrTH was validated with a synthetic peptide of the same sequence (SI, Fig. S17, S18). The shift of 16 Da for the abundant fragment ions around the Pro residue (y”_3_, y”_4_, y”_5_, y”_6,_ b_4_) proves the position of the oxidation on the Pro residue.

### Biological activity of the novel hypertrehalosemic peptides

Once the novel HrTHs were chemically identified, they were commercially synthesised and tested in a heterospecific in vivo biological assay at a concentration of 10 pmol in American cockroaches. An additional octapeptide was synthesised and tested: the peptide has the same amino acid residues as Peram-CAH-I but with a scrambled location of the aromatic amino acids (i.e. Trp^4^ and Phe^8^ instead of Phe^4^ and Trp^8^). Table 3 compares the results of the four novel HrTHs with two positive controls (the endogenous octapeptide Peram-CAH-I and the known blaberid decapeptide Bladi-HrTH) and with two negative controls (water and the Trp^4^Phe^8^-Peram-CAH-I analogue). Injections of the two negative controls had no significant effect on trehalose release from the fat body into the haemolymph, in contrast to all other peptides. The individual hypertrehalosaemic response was calculated as per cent relative change in each group of cockroaches, and the means were subjected to ANOVA analyses with Tukey’s HSD test to interrogate statistically significant differences (Table 3): although there is a clear ranking of the relative biological response from the highest (Peram-CAH-I) to the lowest (Blaat-HrTH) with the cockroach decapeptides tested, these differences are not statistically significant.

**Table 3:** Biological activity of the synthetic equivalent of hypertrehalosaemic hormones synthesised in the corpora cardiaca of *Panchlora nivea*, *Blaberus atropos*, *Eublaberus posticus*, *Episymploce sundaica,* and *Asiablatta kyotensis* in a heterospecific assay with *Periplaneta americana* as acceptor insect

Treatment	*n*	[Carbohydrates]T_0_min (µg/µL)	[Carbohydrates]T_90_min (µg/µL)	Difference (µg/µL)	P*	Relative change (%)**
Distilled water	11	17.68 ± 3.66	17.30 ± 3.91	-0.38 ± 2.34	NS	− 2.07 ± 3.70^a^
Bladi-HrTH (10 pmol) (pQVNFSPGWGT amide)	5	20.38 ± 3.89	36.46 ± 9.41	16.08 ± 6.29	0.002	77.80 ± 9.55^b^
Panni-HrTH (= L^2^-Bladi-HrTH, 10 pmol) (pQLNFSPGWGT amide)	6	19.34 ± 3.50	34.32 ± 5.05	14.98 ± 5.72	0.0007	81.68 ± 16.68^b^
Blaat-HrTH (= L^2^F^10^-Bladi-HrTH, 10 pmol) (pQLNFSPGWGF amide)	9	15.44 ± 3.62	24.10 ± 6.51	8.66 ± 6.67	0.002	61.62 ± 17.56^b^
Lobde-HrTH (= L^2^P^10^-Bladi-HrTH, 10 pmol) (pQLNFSPGWGP amide)	9	17.81 ± 2.06	31.13 ± 6.84	13.32 ± 6.51	0.0001	75.28 ± 12.39^b^
Asiky-HrTH (= L^2^V^10^-Bladi-HrTH, 10 pmol) (pQLNFSPGWGV amide)	8	15.88 ± 2.62	27.47 ± 4.30	11.60 ± 3.35	0.00001	74.80 ± 8.71^b^
W^4^F^8^-Peram-CAH-I (10 pmol) (pQVNWSPNF amide)	6	20.17 ± 1.87	20.71 ± 3.22	0.54 ± 4.30	NS	3.87 ± 8.58^a^
Peram-CAH-I (10 pmol) (pQVNPSPNW amide)	9	18.95 ± 2.94	35.92 ± 4.39	16.97 ± 3.61	0.0000003	91.42 ± 7.89^b^

Distilled water and the synthetic equivalent of one of the endogenous *P. americana* HrTH peptide were injected as control substances, as well as a synthetic peptide that is not known to occur in nature (W^4^F^8^-Peram-CAH-I). Circulating carbohydrates were measured

Data given as mean ± SD, except for relative change, where data are mean ± SE

^*^A paired *t*-test was applied to compare data before and after injection in the same individuals. NS, not significant

^**^Different letters in the column indicate significant differences, Tukey’s HSD test (*p* < 0.05)

## Discussion

A recent survey of genomic/transcriptomic information in publicly accessible databases, for the purposes of phylogenetic analyses based on AKH precursor sequences and AKH receptor sequences in the order Blattodea (termites and cockroaches), revealed seven preprohormone sequences that predicted novel AKH peptides in cockroaches (Jiang et al. 2023). One of the major objectives of the current study was to clarify with sound chemical methods the primary structure of those predicted AKH family peptides and to ascertain the physiological role of those predicted peptides as hypertrehalosaemic hormones (HrTHs) in a well-studied cockroach species, *Periplaneta americana*. We were able to source cockroach species in which four out of the seven peptides should occur. Unfortunately, the remaining three predicted decapeptides were proposed to occur in species that are not in culture in any laboratory, hence not easy to obtain for validating the predicted structures. Due to the key structural features of members of the AKH peptide family, such as the post-translationally modified termini (a pyroglutamic acid at the N-terminus and a carboxyamide at the C-terminus), it is not clear from molecular sequences alone whether during the biosynthesis process of the large precursor peptide, the resulting mature peptide will display the typical AKH family characteristics, or whether the dibasic splicing site is cleaved differently, thereby resulting in a longer C-terminally unblocked entity with negative consequences for functional activation of the AKH receptor (Marco et al. 2022). Likewise, the presence of post-translational modifications of non-terminal amino acids of the AKH/HrTH peptide is potentially possible and not predictable at this stage, for example C-Trp mannosylation (Gäde et al. 1992; Munte et al. 2008), Thr phosphorylation (Gäde et al. 2006), hydroxyprolination (Gäde et al. 2011), or Thr sulphation (Gäde et al. 2014).

**Fig. 8:**
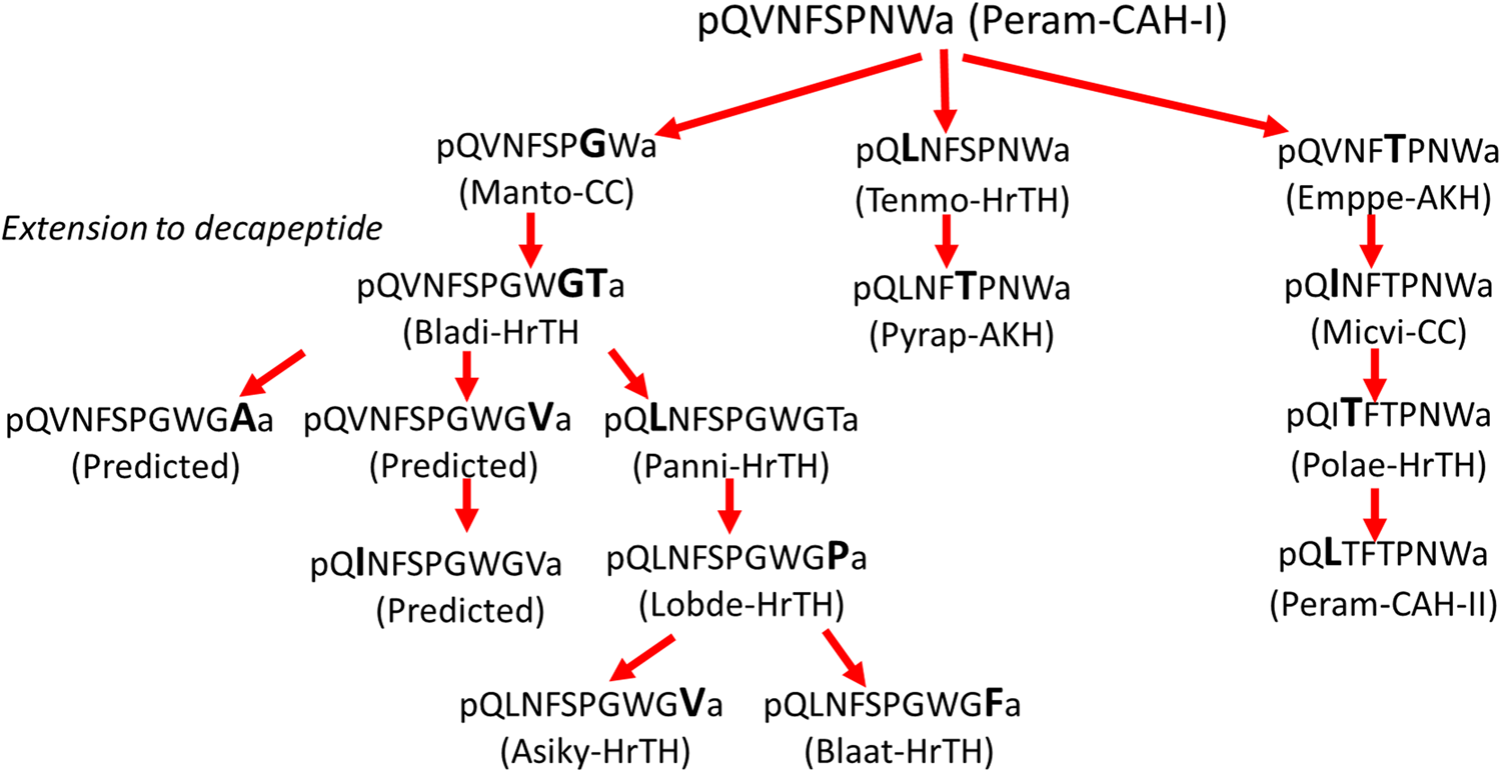
Hypothetical molecular evolution of adipokinetic peptides in Blattodea. Peram-CAH-I is assumed as ancestral peptide for this order. The amino acid substitution in each peptide is indicated in a **bold** larger font than the peptide from which it is hypothetically derived. All substitutions are point mutations except the change from Peram-CAH-I to Manto-CC, where the switch from Asn^7^ to Gly^7^ requires two base changes. Note that three hitherto unconfirmed novel decapeptides (= Predicted, from data mining databases of *Cariblatta* sp. and *Nyctibora* sp.; see Jiang et al. 2023) are included in the scheme

The MS measurements of the current study gave clear answers to the questions of the structure of four of the predicted novel peptides, which we have now code-named Panni-HrTH (in *Panchlora nivea*), Blaat-HrTH (in *Blaberus atropos*), Lobde-HrTH (in *Loboptera decipiens* and *Episymploce sundaica*), and Asiky-HrTH (in *Asiablatta kyotensis* and *Supella longipalpa*). We could neither confirm nor refute the primary structure of the three remaining novel peptides as we do not have access to the cockroach species in which the peptides were predicted from transcriptomes, viz. in *Cariblatta* sp. and *Nyctibora* sp. (Jiang et al. 2023). All 13 investigated cockroach species in the current study produce HrTHs that have the characteristic post-translationally modified termini of this peptide family. Furthermore, the peptides are correctly cleaved at the dibasic splicing site. The MS technique also revealed unequivocally that the genomically/transcriptomically predicted forms of HrTHs in the investigated blaberoid cockroaches are indeed expressed and confirmed the sequences. Validation of the natural and synthetic compounds by retention time and CID pattern resulted always in a perfect match. Thus, there is no doubt that certain blaberid cockroaches produce two decapeptides in their CC, both of which are very similar in primary structure and could have evolved from each other. A hypothetical scheme for molecular evolution of all Blattodea HrTHs is given in Fig. 8. It is noteworthy that all decapeptides, including the three that are still only predicted (see Fig. 8), are on one side of the scheme and can evolve from each other with only point mutations. Only in one case of the Blattodea HrTH peptides, involving the octapeptides Peram-CAH-I and Manto-CC, would two nucleotides of the triplet have to mutate in this hypothetical evolutionary scheme.

In the majority of cockroach samples analysed in the current study, the crude extracts of corpora cardiaca displayed an additional mass signal that when sequenced, corresponded to the hypertrehalosaemic peptide(s) of that species with a hydroxyproline residue at position 6; we have validated only one of these sequences ([Hyp^6^]-Tenmo-HrTH, while the other “putatives” remain to be validated. In insects, hydroxyprolination of an AKH was first shown in the green stink bug *Nezara viridula* (order Hemiptera, suborder Heteroptera) as an analogue of the octapeptide Panbo-RPCH (Gäde et al. 2011), and recently for Tabat-AKH in the horse fly *Haematopota pluvialis* (Haepl-AKH; Marco et al. 2022). In *N. viridula,* it was shown that Nezvi-AKH—the peptide with the hydroxyproline in position 6—was a genuine occurrence and not merely an artefact; furthermore, this atypical AKH was biologically active in lipid mobilisation in the stink bug, as was its Pro analogue, Panbo-RPCH (Gäde et al. 2011). Possibly, hydroxyprolination of AKHs is more common than previously thought, and the enigma of whether this modification may be a natural phenomenon in the CC, or an artefact arising during peptide handling will require further investigation.

Another objective of this study was to show that the novel decapeptides have biological, thus trehalose mobilising activity in cockroaches. In most of the investigated species, the decapeptide Bladi-HrTH (pQVNFSPGWGT amide) was detected and validated (Table 1), adding to the body of information that this is a hallmark peptide of blaberid cockroaches (see, e.g., Hayes et al. 1986; Gäde and Rinehart 1986; Roth et al. 2009; Jiang et al. 2023). Although the American cockroach is a blattid cockroach, we nevertheless selected it as acceptor insect for in vivo functional assays, for two reasons: haemolymph of *P. americana* does not clot easily, and there is a plethora of physiological information on this species, including structure–activity studies with the AKH family of peptides (see Introduction). *P. americana* synthesises two octapeptides, but its AKH receptor also recognises the decapeptide Bladi-HrTH very well: whereas the endogenous hormones Peram-CAH-I and Peram-CAH-II had ED_50_ values (the amount of peptide needed to produce 50% of the maximal possible response) between 0.5 and 1 pmol, a maximum response was achieved with 4 pmol, the ED_50_ value for Bladi-HrTH was between 1.6 and 3 pmol, and 10 pmol were needed for a maximum response (Gäde 1990; Gäde and Hayes 1990; Table 3). The first six amino acids of Bladi-HrTH are identical with those of Peram-CAH-I with a difference occurring at position 7, viz. a simple glycine residue instead of the asparagine of the *P. americana* octapeptide (see Table 1 for structures). Peram-CAH-II, the second HrTH octapeptide of *P. americana* on the other hand, registers three differences in the first six amino acids compared with Peram-CAH-I and Bladi-HrTH and still elicited a 100% hypertrehalosaemic response in *P. americana* (Gäde 1990). The novel cockroach decapeptides validated in this study may be described as analogues of Bladi-HrTH for they differ from Bladi-HrTH in only one or two positions; they are also able to activate the *P. americana* HrTH receptor (Table 3). The leucine at position 2 (Panni-HrTH) is well tolerated, which is in line with a leucine residue at this position in the second *P. americana* octapeptide.

A double substitution compared to Bladi-HrTH, leucine at position 2 plus either valine, proline or phenylalanine at position 10 instead of threonine produced smaller trehalosaemic responses than measured with Bladi-HrTH and Panni-HrTH but did not differ significantly from the increase resulting from Peram-CAH-I (Table 3). It would seem, thus, that although the *P. americana* HrTH receptor favours octapeptides, decapeptides can bind and influence the biological activity in this blattid cockroach. Structure–activity studies have also been reported for *B. discoidalis* with Bladi-HrTH analogues (Ford and Hayes 1988; Hayes and Keeley 1990): Ala^2^-Bladi-HrTH and Ala^10^-Bladi-HrTH had small or no influence on activity. Thus, it is very likely that the novel peptides which were confirmed by MS methods in this study may also have no or only little influence on binding to the *B. discoidalis* HrTH receptor and will perform with maximal activity in blaberid cockroaches.

Ideally, the novel peptides should also be tested conspecifically. This, however, is not such a simple task, because the haemolymph of blaberid cockroaches coagulates immediately when in contact with air. A method is described and evaluated to determine trehalose in the coagulum of *B. discoidalis* (Keeley et al. 1991; Sowa and Keeley 1996). If this method can be modified for small species of the genera *Episymploce*, *Asiablatta*, *Supella* and *Panchlora* remains to be seen.

Lastly, this study may also have helped in the discussion of phylogenetic placement of certain cockroach families. Whereas Legendre et al. (2015) have *Anaplecta* in the superfamily Blaberoidea nested within Ectobiidae as sister taxon to several Pseudophyllodromiinae, Djernæs et al. (2020) have this species and family Anaplectidae in the superfamily Blattoidea close to the clades Tryonicidae, Lamproblattidae, and Cryptocercidae plus Isoptera (termites). Our finding of Peram-CAH-I in *Anaplecta* is consistent with the latter view.

### Supplementary Information

Below is the link to the electronic supplementary material.

Supplementary file1 (DOCX 4250 KB)

## Data Availability

All data generated or analysed during this study are included in this published article [and its supplementary information files].

## Abbreviation

HrTH: Hypertrehalosaemic hormone
AKH: Adipokinetic hormone
CC: Corpora cardiaca
HPLC: High-performance liquid chromatography
MS: Mass spectrometry
RP-LC: Reversed-phase liquid chromatography
