# Supplementary material for: Predicted novel hypertrehalosaemic peptides of cockroaches are verified by mass spectrometry
Source: Amino Acids. 2023 Oct 26;55(11):1641–54. doi: 10.1007/s00726-023-03337-7 (PMC10689539; doi:10.1007/s00726-023-03337-7)
Supplement: Supplementary file 1 — Supplementary file1 (DOCX 4250 KB) [file 726_2023_3337_MOESM1_ESM.docx]

**Predicted novel hypertrehalosaemic peptides of cockroaches are verified by mass spectrometry**

Heather G. Marco^1^, Simone König^2#^ and Gerd Gäde^1* #^

^1^Department of Biological Sciences, University of Cape Town, Rondebosch, Cape Town, South Africa; gerd.gade@uct.ac.za; heather.marco@uct.ac.za

^2^IZKF Core Unit Proteomics, University of Münster, Münster, Germany; koenigs@uni-muenster.de

^#^ These authors contributed equally to this paper

*****Correspondence: gerd.gade@uct.ac.za; Tel.: +27 21 650 3615

**Amino Acids: Supplementary Information**

Here, we present raw spectra and explanatory data as mentioned in the main manuscript. We show the expected ions for sequence hypotheses. For instance, in Fig. S1, the expected fragment ions for sequence pQVNFSPGWGTa (blocked termini: pQ and amidation, = Bladi-HrTH) are given as generated by MassLynx software. Shown are the b- and the y”- ion series with the corresponding neutral water and ammonia losses (y~, b~, y*, b*) as well as the immonium ions (i). These ions have to match peaks in the spectra such as 516.257 (y”_5_) and 899.405 (b_7_) in Fig. S2. The spectra represent target analyses on the singly (M+H^+^)- and/or doubly-charged )[M+2H]^2+^ ions of a certain AKH.


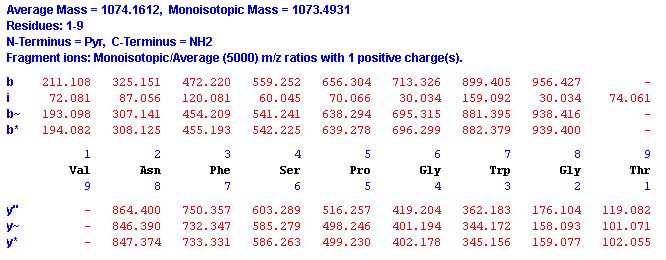


**Figure S1.** Calculation of the expected fragment ions for sequence pQVNFSPGWGTa (= Bladi-HrTH) using MassLynx (blocked termini: pQ and amidation).

A)


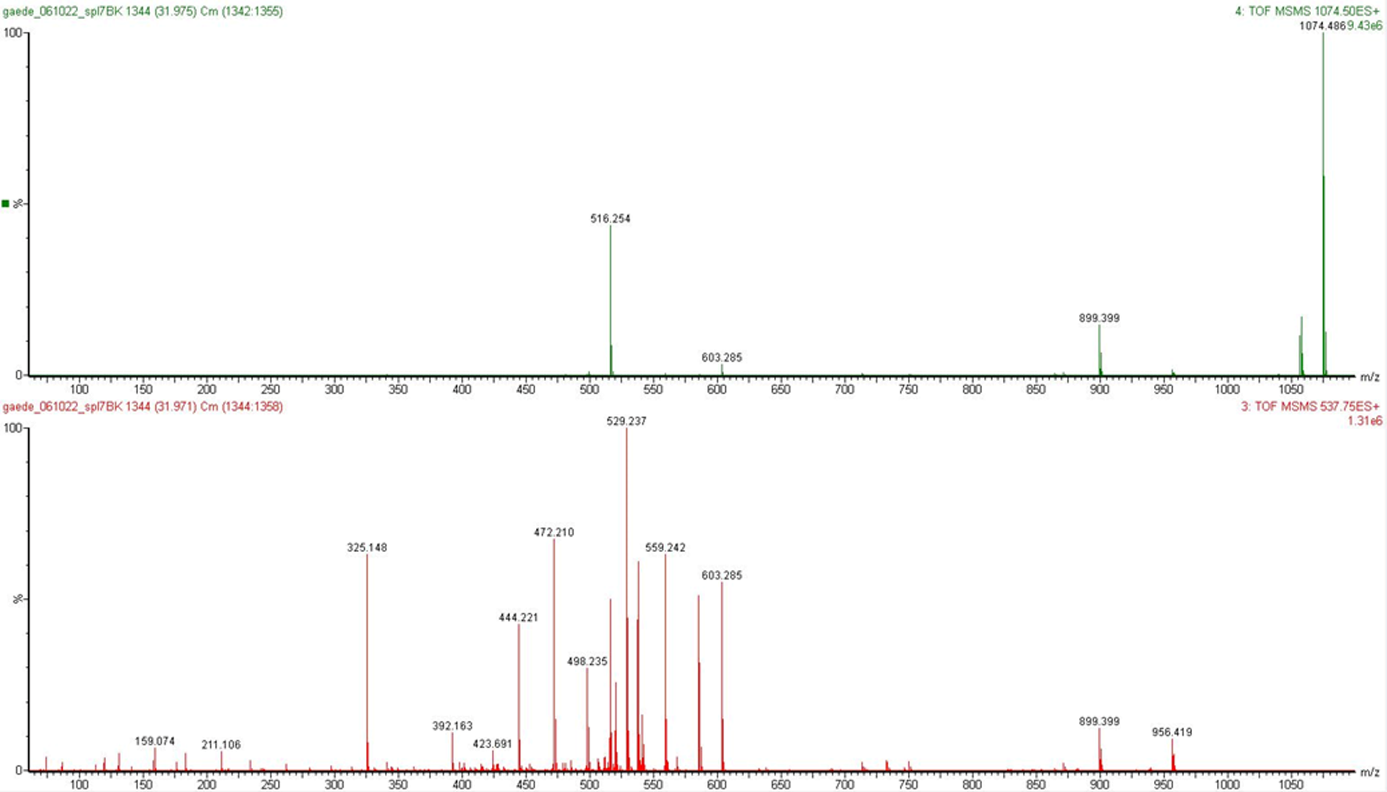


B)

**
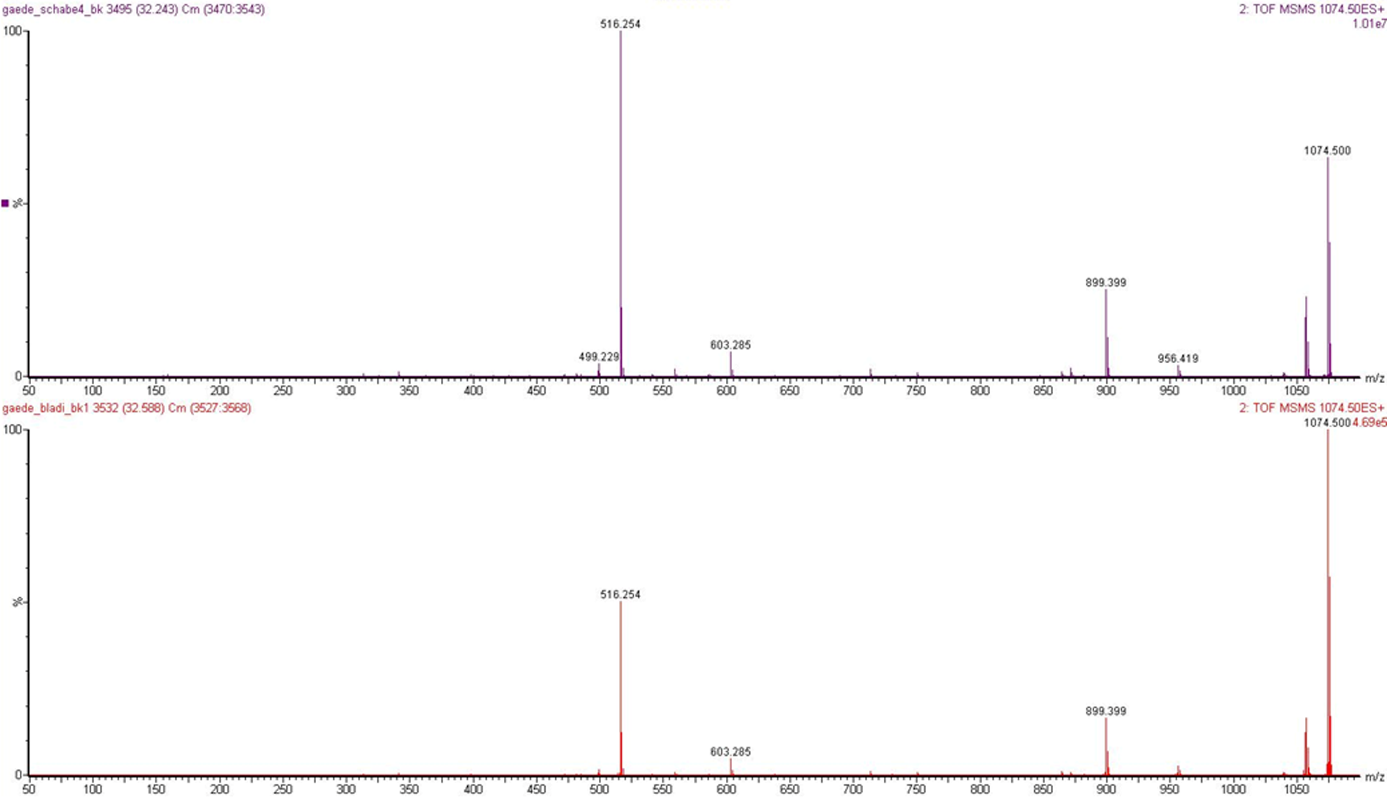
**

**Figure S2.** Validation of Bladi-HrTH (pQVNFSPGWGTa) in A) *Panchlora nivea* (MS/MS of M+H^+^ 1074.50 and [M+2H]^2+^ 537.75, B) *Xestoblatta cavicola* (top panel, MS/MS of M+H^+^ 1074.50)*.* B) Bottom panel: Bladi-HrTH reference spectrum measured with synthetic standard (MS/MS of M+H^+^ 1074.50). Blocked termini: pQ and amidation.


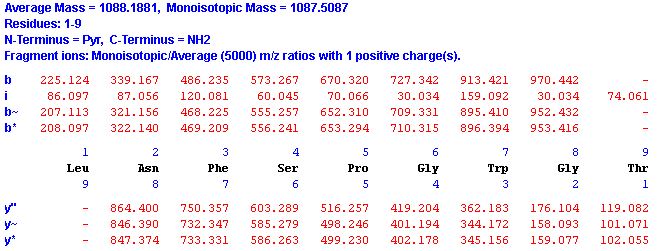


**Figure S3.** Calculation of the expected fragment ions for sequence pQLNFSPGWGTa (= Panni-HrTH) using MassLynx (blocked termini: pQ and amidation).


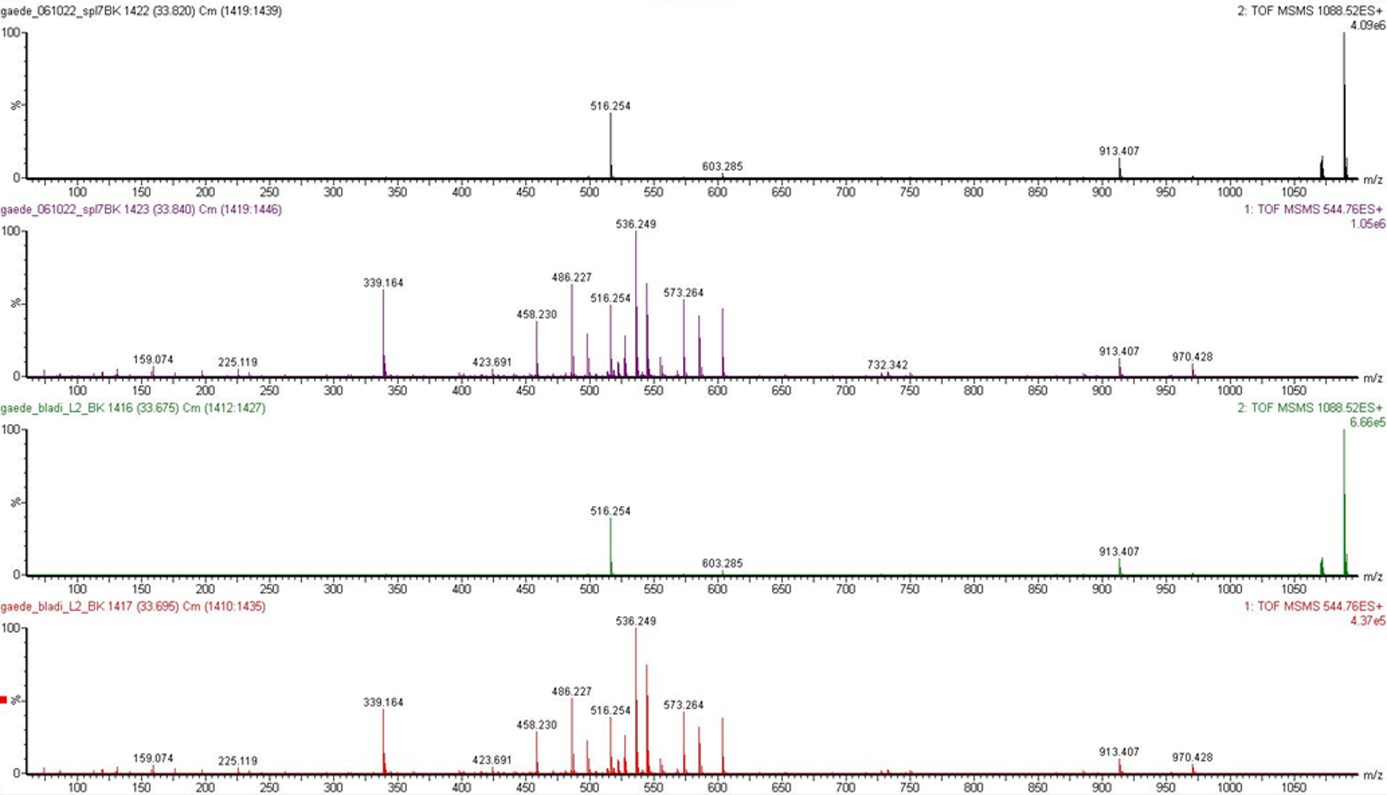


**Figure S4.** Validation of Panni-HrTH (pQLNFSPGWGTa) in *Panchlora nivea* (top two panels). MS/MS of the singly- (M+H^+^ 1088.52) and the doubly-charged ([M+2H]^2+^ 544.76) ions. Bottom two panels: Panni-HrTH reference spectra measured with synthetic standard. Blocked termini: pQ and amidation.


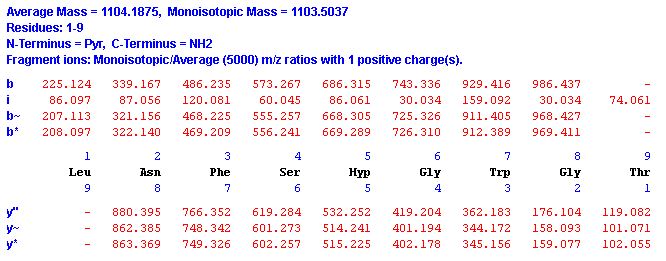


**Figure S5.** Calculation of the expected fragment ions for sequence pQLNFSP(Hyp)GWGTa of a peptide assigned to [Hyp^6^]-Panni-HrTH using MassLynx (blocked termini: pQ and amidation).

**
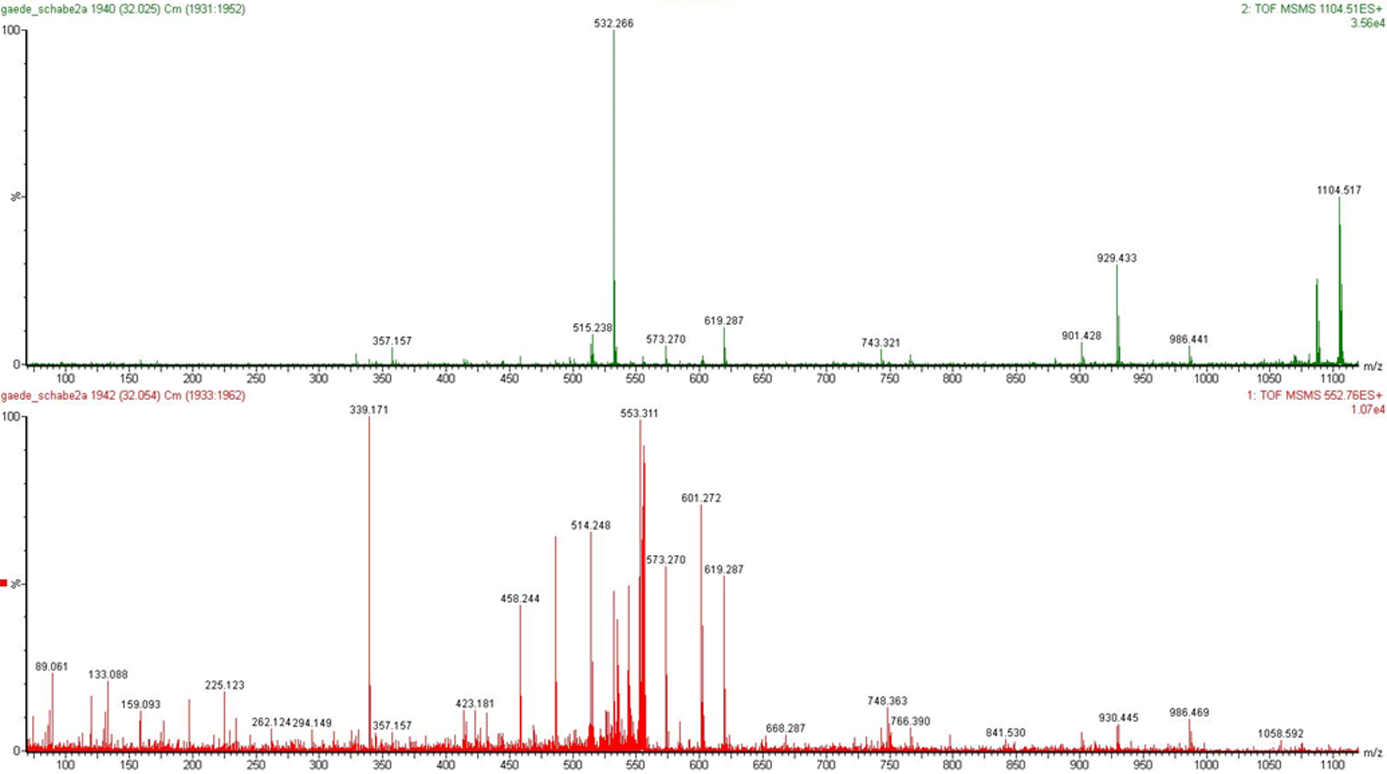
**

**Figure S6.** MS/MS analysis for the singly- and the doubly-charged peptide ions (*m/z* 1104.51 and 552.76) of a peptide assigned to [Hyp^6^]-Panni-HrTH in *Panchlora nivea*.


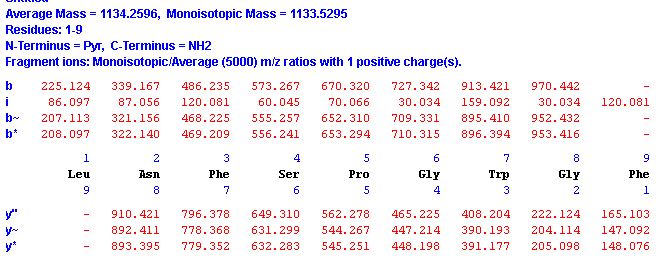


**Figure S7.** Calculation of the expected fragment ions for sequence pQLNFSPGWGFa (= Blaat-HrTH) using MassLynx (blocked termini: pQ and amidation).

**
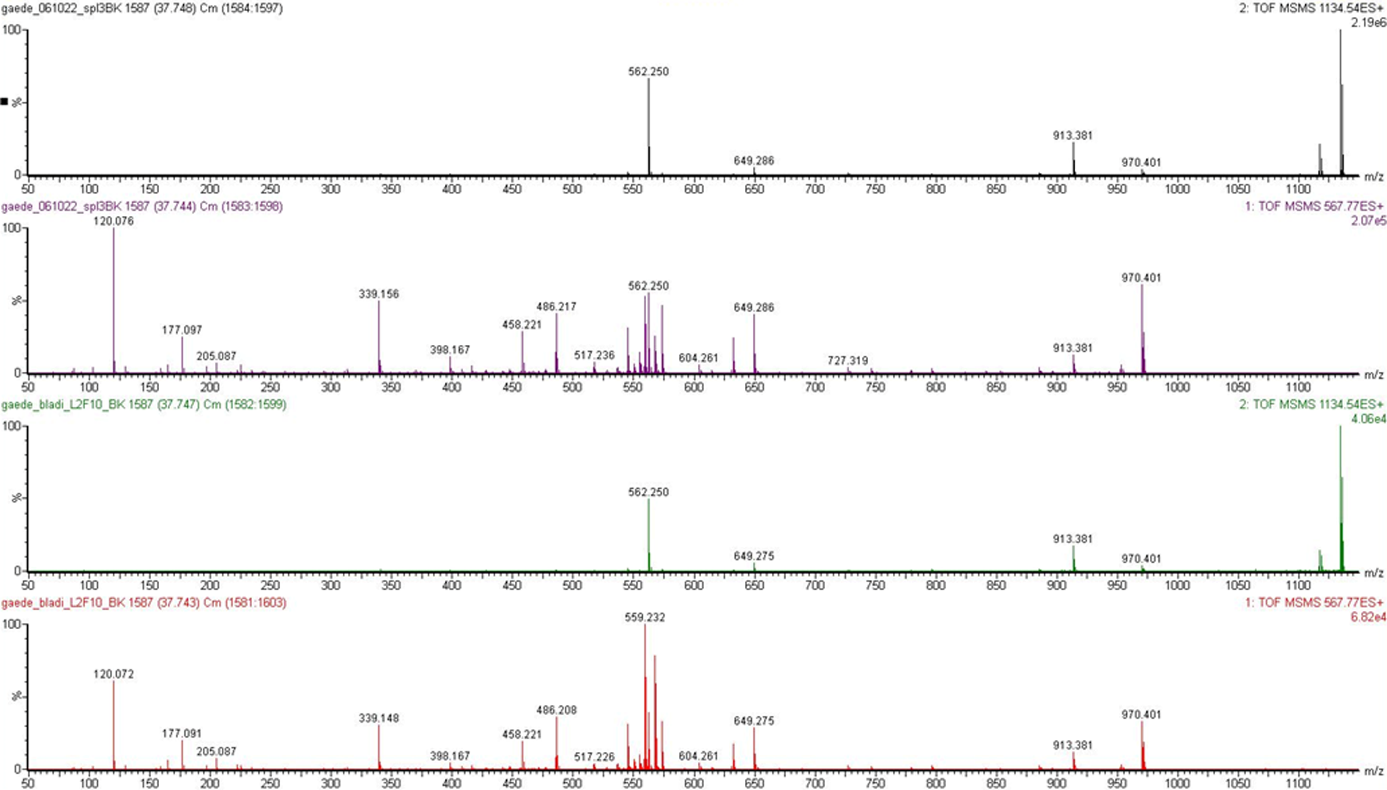
**

**Figure S8.** Validation of Blaat-HrTH (pQLNFSPGWGFa) in *Blaberus atropos*. MS/MS of the singly- (M+H^+^ 1134.54) and the doubly-charged ([M+2H]^2+^ 567.77) ions. Bottom two panels: Blaat-HrTH reference spectra measured with synthetic standard. Blocked termini: pQ and amidation.


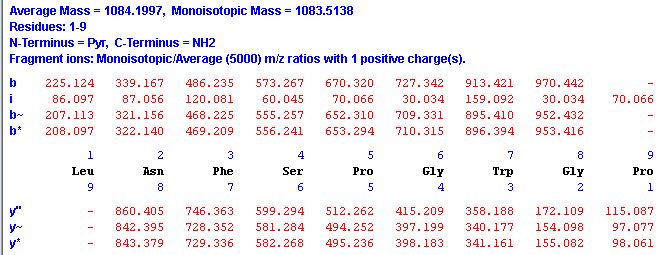


**Figure S9.** Calculation of the expected fragment ions for sequence pQLNFSPGWGPa (= Lobde-HrTH) using MassLynx (blocked termini: pQ and amidation).

**
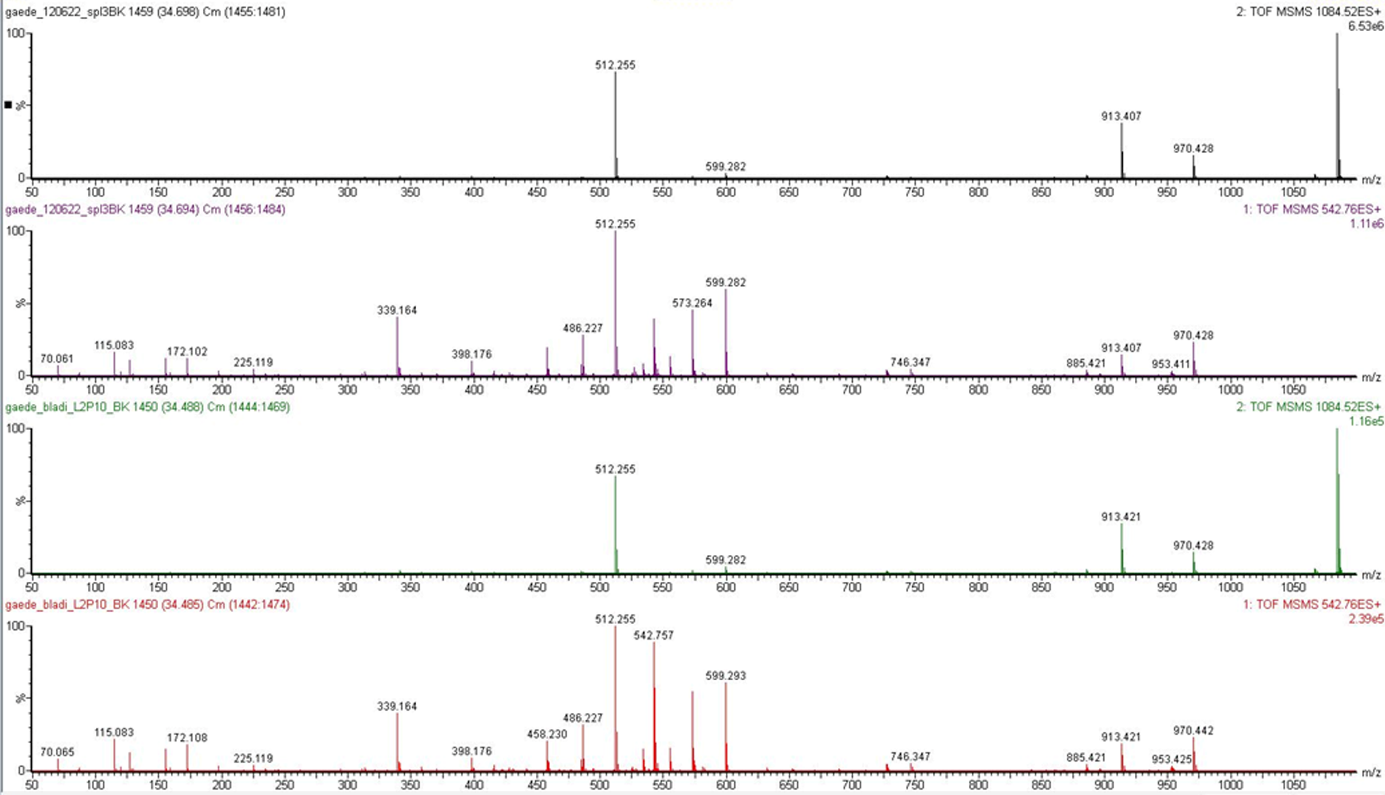
**

**Figure S10.** Validation of Lobde-HrTH (pQLNFSPGWGPa) in *Loboptera decipiens*. MS/MS of the singly- (M+H^+^ 1084.52) and the doubly-charged ([M+2H]^2+^ 542.76) ions. Bottom two panels: Lobde-HrTH reference spectra measured with synthetic standard. Blocked termini: pQ and amidation.


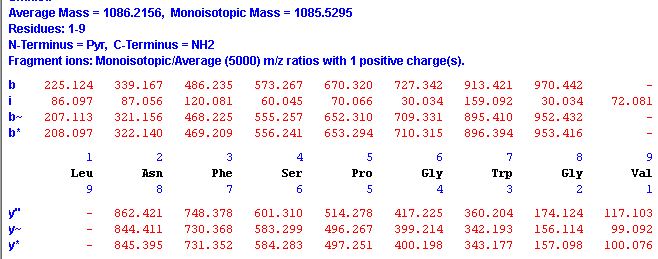


**Figure S11.** Calculation of the expected fragment ions for sequence pQLNFSPGWGVa (= Asiky-HrTH) using MassLynx (blocked termini: pQ and amidation).

**
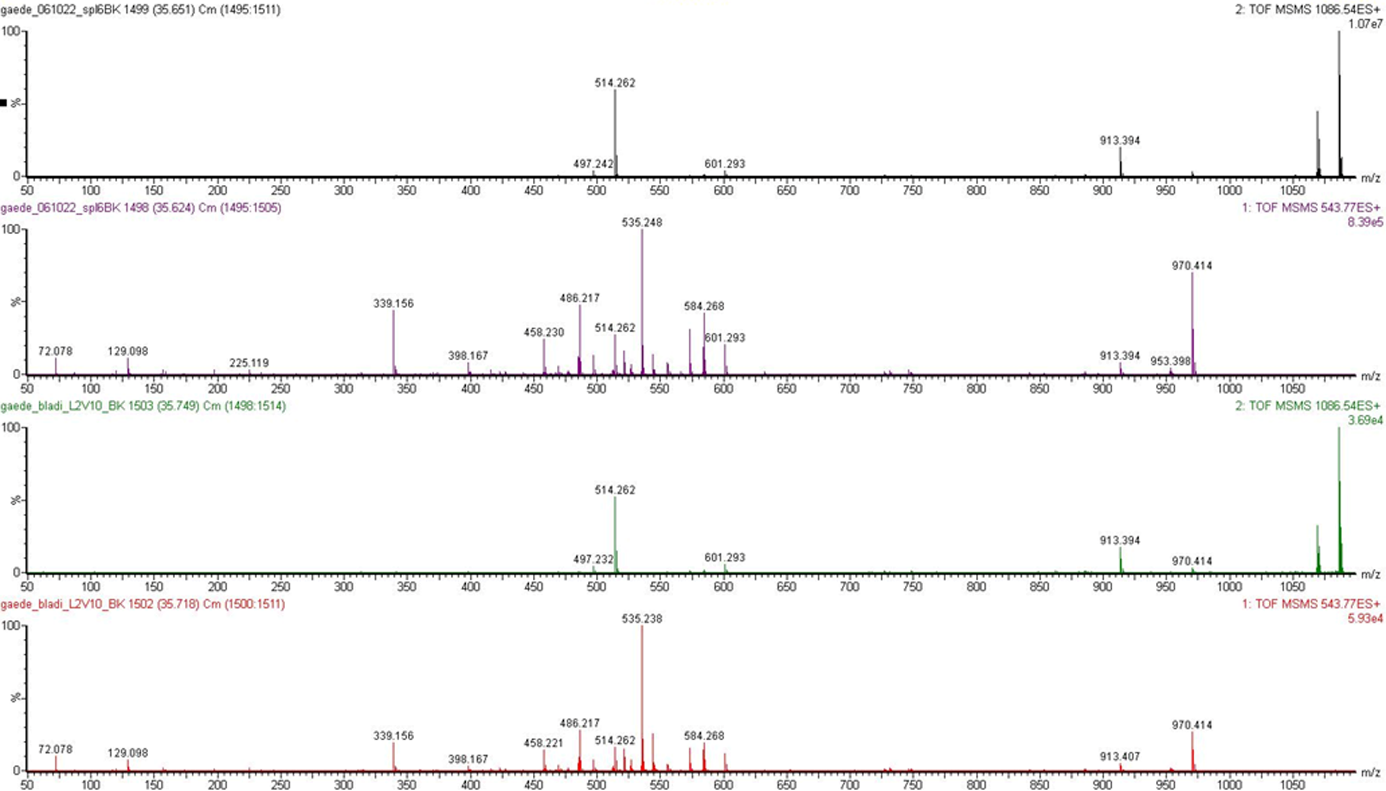
**

**Figure S12.** Validation of Asiky-HrTH (pQLNFSPGWGVa) in *Asiablatta kyotensis*. MS/MS of the singly- (M+H^+^ 1086.54) and the doubly-charged ([M+2H]^2+^ 543.77) ions. Bottom two panels: Asiky-HrTH reference spectra measured with synthetic standard. Blocked termini: pQ and amidation.


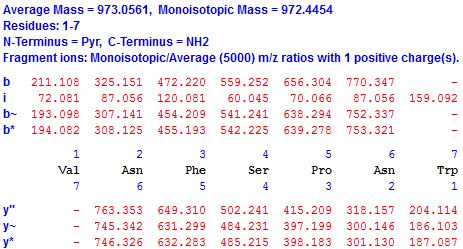


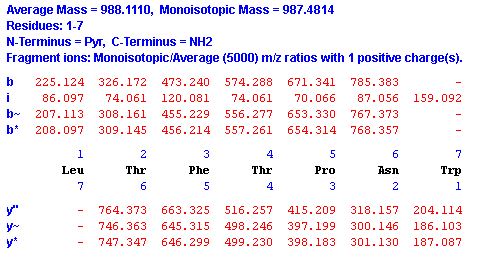


**Figure S13.** Calculation of the expected fragment ions for sequences pQVNFSPNWa (= Peram-CAH-I) and pQLTFTPNWa (= Peram-CAH-II) using MassLynx (blocked termini: pQ and amidation).

A)

**
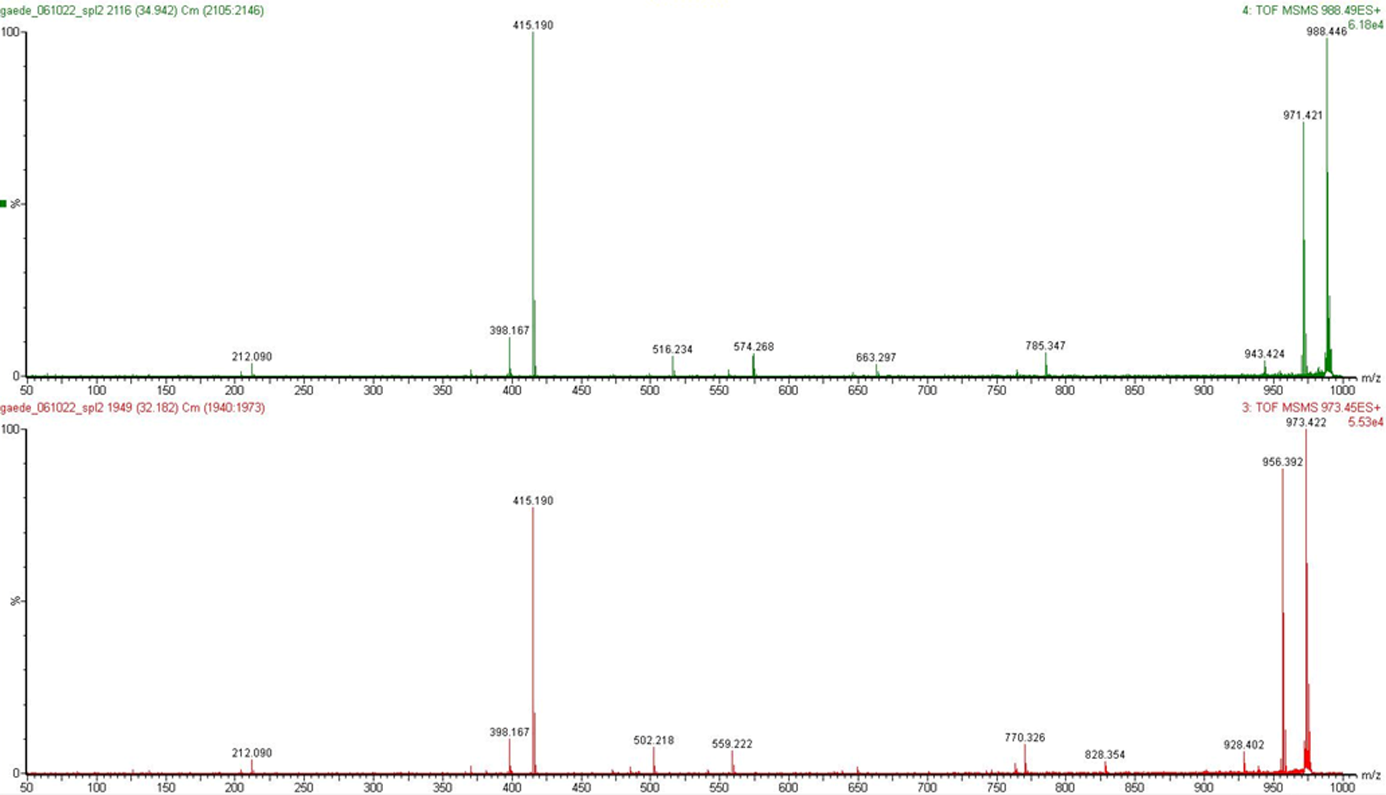
**

B)


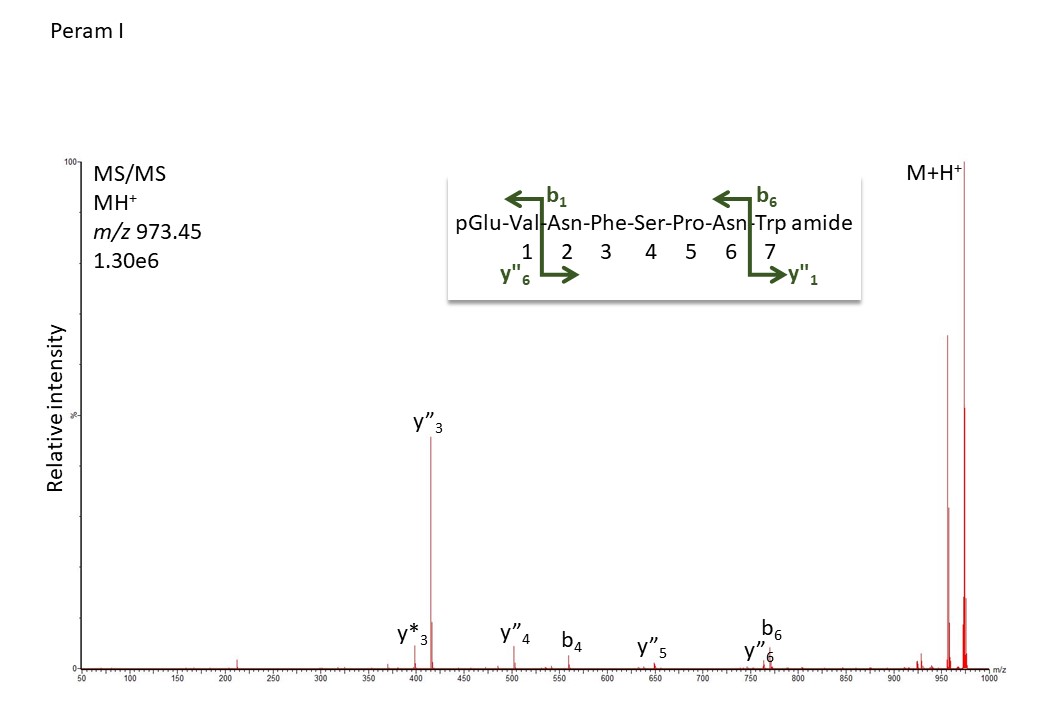


**Figure S14.** MS/MS spectra of the singly-charged ions for Peram CAH-I (M+H^+^ 988.49) and -II (*m/z* 973.45) in A) *Periplaneta americana* and B) Peram CAH-I in *Anaplecta ssp.* Blocked termini: pQ and amidation.


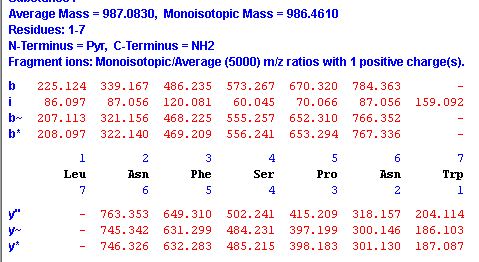


**Figure S15.** Calculation of the expected fragment ions for sequence pQLNFSPNWa (= Tenmo-HrTH) using MassLynx (blocked termini: pQ and amidation).


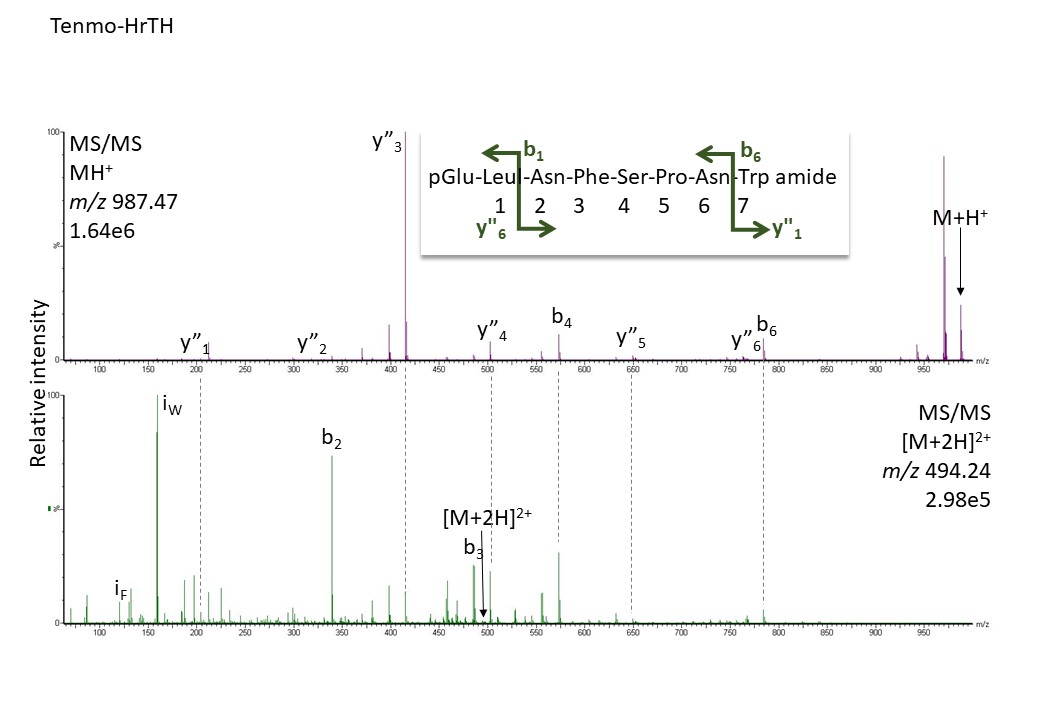


**Figure S16.** MS/MS spectra of the singly- (upper panel) and the doubly-charged (lower panel) ions of Tenmo-HrTH (pQLNFSPNWa) in *Ergaula capucina*. Blocked termini: pQ and amidation.


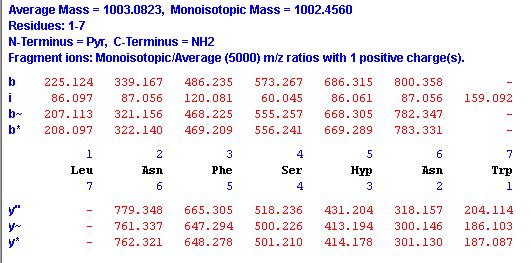


**Figure S17.** Calculation of the expected fragment ions for sequence pQLNFSP(Hyp)NWa of a peptide assigned to [Hyp^6^]-Tenmo-HrTH using MassLynx (blocked termini: pQ and amidation).


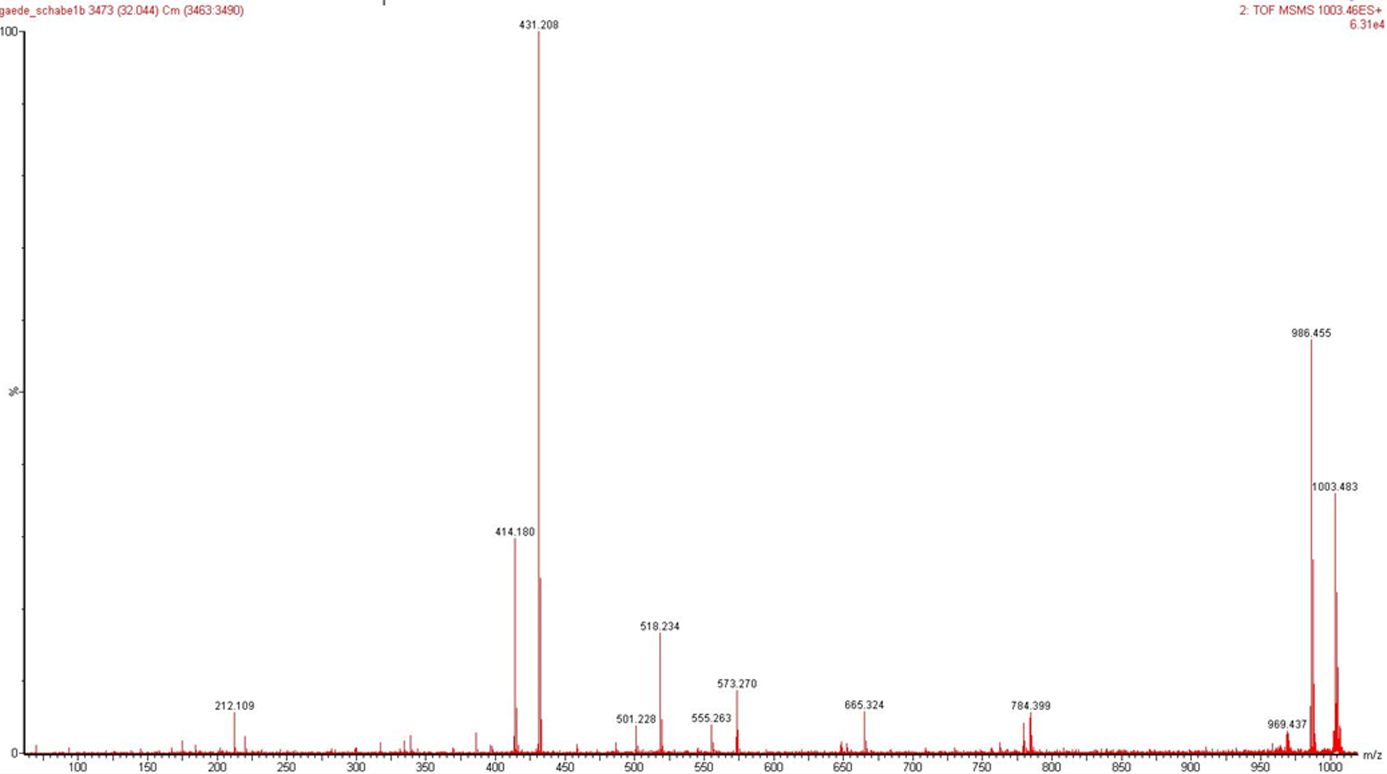


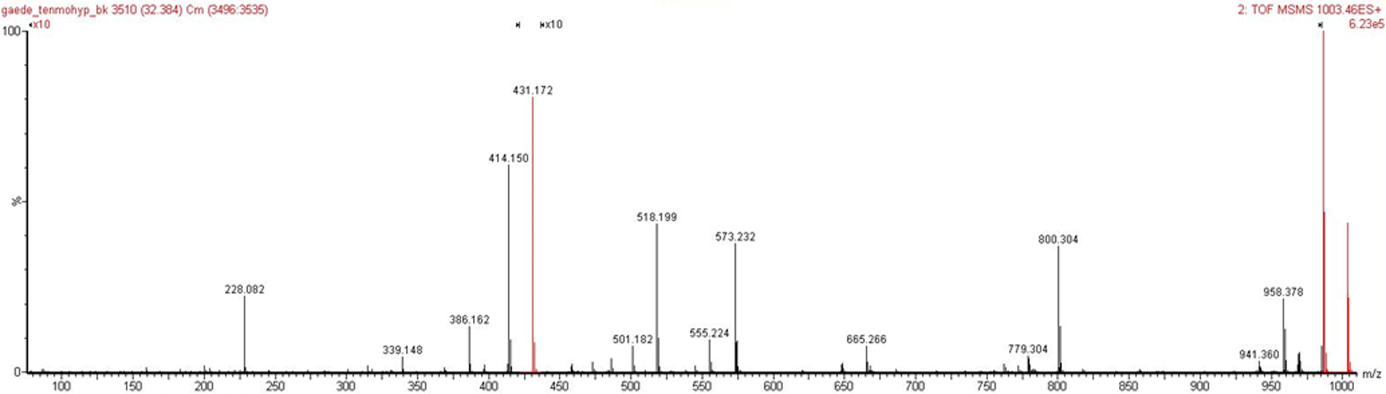


**Figure S18.** MS/MS spectrum of the singly-charged ion of a peptide assigned to [Hyp6]-Tenmo-HrTH in *Ergaula capucina* and its validation with the synthetic compound (bottom spectrum).
